# Optimisation of Solid-Phase Extraction and LC-MS/MS Analysis of Six Breast Cancer Drugs in Patient Plasma Samples

**DOI:** 10.3390/ph16101445

**Published:** 2023-10-12

**Authors:** Lu Turković, Dragana Mutavdžić Pavlović, Zvonimir Mlinarić, Anamarija Skenderović, Tajana Silovski, Miranda Sertić

**Affiliations:** 1 Department of Pharmaceutical Analysis, Faculty of Pharmacy and Biochemistry, University of Zagreb, Ante Kovacica 1, 10000 Zagreb, Croatiazvonimir.mlinaric@pharma.unizg.hr (Z.M.); 2 Department of Analytical Chemistry, Faculty of Chemical Engineering and Technology, University of Zagreb, Marulicev trg 20, 10000 Zagreb, Croatia; dmutavdz@fkit.hr; 3 GxR&D Analytics Zagreb, Global R&D, Teva Pharmaceuticals, Prilaz Baruna Filipovica 25, 10000 Zagreb, Croatia; anamarija.skenderovic@pliva.com; 4 Department of Oncology, University Hospital Centre Zagreb, Kispaticeva 12, 10000 Zagreb, Croatia; tsilovsk@kbc-zagreb.hr; 5 School of Medicine, University of Zagreb, Salata 3, 10000 Zagreb, Croatia

**Keywords:** solid-phase extraction, breast cancer, CDK4/6 inhibitors, therapeutic drug monitoring, liquid chromatography, mass spectrometry, bioanalysis, patient plasma

## Abstract

In the development of bioanalytical LC-MS methods for the determination of drugs in plasma samples in a clinical setting, adequate sample preparation is of utmost importance. The main goals are to achieve the selective extraction of the analytes of interest and attain thorough matrix removal while retaining acceptable ecological properties, cost-effectiveness, and high throughput. Solid-phase extraction (SPE) offers a versatile range of options, from the selection of an appropriate sorbent to the optimisation of the washing and elution conditions. In this work, the first SPE method for the simultaneous extraction of six anticancer drugs used in novel therapeutic combinations for advanced breast cancer treatment—palbociclib, ribociclib, abemaciclib, anastrozole, letrozole, and fulvestrant—was developed. The following sorbent chemistries were tested: octylsilyl (C8), octadecylsilyl (C18), hydrophilic–lipophilic balance (HLB), mixed-mode cation-exchange (MCX and X-C), and mixed-mode weak cation-exchange (WCX), with different corresponding elution solvents. The samples were analysed using LC-MS/MS, with a phenyl column (150 × 4.6 mm, 2.5 μm). The best extraction recoveries (≥92.3%) of all analytes were obtained with the C8 phase, using methanol as the elution solvent. The optimised method was validated in the clinically relevant ranges, showing adequate precision (inter-day RSD ≤ 14.3%) and accuracy (inter-day bias −12.7–13.5%). Finally, its applicability was successfully proven by the analysis of samples from breast cancer patients.

## 1. Introduction

Palbociclib (PAL), ribociclib (RIB), and abemaciclib (ABE) are newly registered anticancer drugs, inhibitors of the cyclin D-dependent kinases 4 and 6 (CDKi). They are prescribed for the treatment of hormone receptor-positive and human epidermal growth factor 2-negative (HR+, HER2−) breast cancer, in synergistic combinations with endocrine therapy (ET): aromatase inhibitors anastrozole (ANA) or letrozole (LET), or an oestrogen receptor antagonist fulvestrant (FUL). It has been shown that, of all HR+, HER2− patients in recent years (2018–2022), about 70–80% were prescribed with CDKi + ET, while chemotherapy was used in about 15% and ET alone in about 10% of the cases [1]. Therapeutic drug monitoring (TDM) of anticancer drugs has great potential in reducing adverse events and improving treatment outcomes for individual patients [2]. The development of an efficient, cost-effective, and ecologically favourable analytical method for the determination of drugs in biological fluids is the first prerequisite for TDM. Simultaneous determination of several drugs with the same analytical conditions would offer better sample turnaround and higher laboratory efficiency, which leads to a faster and more convenient delivery of the results, aiding the subsequent medical decision-making process, as well as having a favourable ecological impact [3].

The six drugs of interest have a wide array of physical-chemical properties, with the CDKi being weak bases, aromatase inhibitors being neutral and relatively hydrophilic, and FUL being highly lipophilic [4,5]. Thus, simultaneous extraction of all six drugs using the same extraction conditions may be challenging. 

Solid-phase extraction (SPE) is a popular option for the preparation of various types of samples, including human plasma—the most commonly used sample in TDM. Its selectivity, purification efficacy, versatility, and ease of automation are among the most prominent advantages that make it suitable for complex bioanalytical sample preparation [6].

The most common commercially available SPE sorbents are either silica- or polymer-based, with different functional groups and retention mechanisms, such as reversed-phase sorbents similar to chromatographic stationary phases—C18, C8, phenyl, etc.; polymeric hydrophilic–lipophilic balance (HLB) sorbents, comprised of polar pyrrolidone and non-polar divinylbenzene; and ion-exchange sorbents containing ionisable groups, such as the sulfonic and carboxylic groups for cation exchange, or amines for anion exchange [7,8,9]. To achieve the simultaneous extraction of both ionisable and non-ionisable analytes with several interaction mechanisms, mixed-mode sorbents containing ion-exchange groups together with the HLB backbone have been introduced. Apart from the choice of sorbent structure, different elution conditions can further fine-tune the SPE performance, while various washing conditions can enhance sample purification and matrix removal [10].

Thus far, several bioanalytical methods for the determination of ABE, PAL, RIB, ANA, LET, and FUL in human plasma samples have been reported. Most of the methods were developed only for selected combinations of the analytes, with three works focused on all six drugs of interest, applying protein precipitation (PPT) and dispersive liquid–liquid microextraction (DLLME) for sample preparation [5,11,12]. For specific analyte combinations, the most common sample preparation procedure applied was PPT with an organic solvent [13,14,15,16,17,18,19,20], liquid–liquid extraction (LLE) [21,22], and SPE [14,23,24,25]. Of the SPE methods applied for some of the analytes of interest, an Oasis HLB sorbent in combination with methanol (MeOH) as the elution solvent was found suitable for the CDKi [24,25], as well as a Phenomenex C18 sorbent eluted with acetonitrile (ACN) following PPT [14]. Aromatase inhibitors were, on the other hand, successfully extracted using a mixed-mode weak cation-exchange sorbent, Strata X-C, eluted with 5% NH_4_OH in MeOH [23]. The structural formulae, as well as important physical-chemical descriptors of the six analytes, are shown in Table 1 [26,27]. Functional groups with the potential for certain intermolecular interactions, which may be important for the retention of different sorbents, are tentatively highlighted. Additional van der Waals forces stemming from dipoles and induced dipoles have not been specifically highlighted but are assumed to be present as well.

To the best of our knowledge, there is no reported protocol for SPE of FUL or a combination of the CDKi with the aromatase inhibitors or FUL from any type of sample thus far. Therefore, the aim of this work was to achieve simultaneous extraction and cleanup with satisfactory extraction yields of all six analytes of interest on a single SPE sorbent. 

## 2. Results and Discussion

### 2.1. Optimisation of the SPE Procedure

Several different SPE sorbents were tested: reversed-phase: octylsilyl (C8, Waters Sep-Pak Vac), octadecylsilyl (C18, Waters Sep-Pak Vac), and hydrophilic–lipophilic balance (HLB, Waters Oasis); ion-exchange sorbents: mixed-mode cation-exchange (MCX, Waters Oasis, and X-C, Phenomenex Strata), and weak cation-exchange (WCX, Waters Oasis).

Reversed-phase sorbents were selected as the most widely applicable sorbents suitable for various types of compounds. Cation-exchange and mixed-mode cation-exchange sorbents were tested due to the basic nature of some of the analytes. Some of these sorbents have already been applied in the literature, but only for the extraction of up to two of the mutually similar drugs of interest. 

In this phase, all samples were analysed using high-performance liquid chromatography equipped with a diode array and fluorescence detection (HPLC-DAD-FLD). This technique was found suitable for the preliminary experiments due to its simplicity, relatively low cost of operation, and the ability to observe the chromatographic profile of the leftover plasma interferences, thus enabling an easier and broader evaluation of the sample cleanup efficiency.

The extraction recoveries were calculated as the ratio of peak areas in spiked plasma samples and standard solutions of the same concentration and are shown in figures as the median and range. Two spiked samples and one blank plasma sample were prepared for each tested condition. Blank sample chromatograms were inspected for matrix interferences at the retention times of the analytes.

#### 2.1.1. Reversed-Phase Sorbents

The structures of the tested reversed-phase and HLB sorbents, with the tentatively highlighted groups responsible for potential intermolecular interactions, are shown in Table 2 [7].

For the experiments, plasma samples were diluted with Milli-Q water in the volume ratio of 4:5. The sorbent-conditioning step included loading with MeOH and Milli-Q water, as per the manufacturer’s instructions [7]. After the application of the sample, a washing step with Milli-Q water and 5% MeOH in water was performed in order to remove the unretained matrix interferences, such as plasma proteins. Different solvents were tested for analyte elution: MeOH and ACN, as organic solvents with different elution strengths, as well as acidified and alkalised MeOH, to test the effect of the pH. Varying the volumes of MeOH was also assessed for the C8 sorbent. All the conditions are summarised in Table 3. 

**Figure 1:**
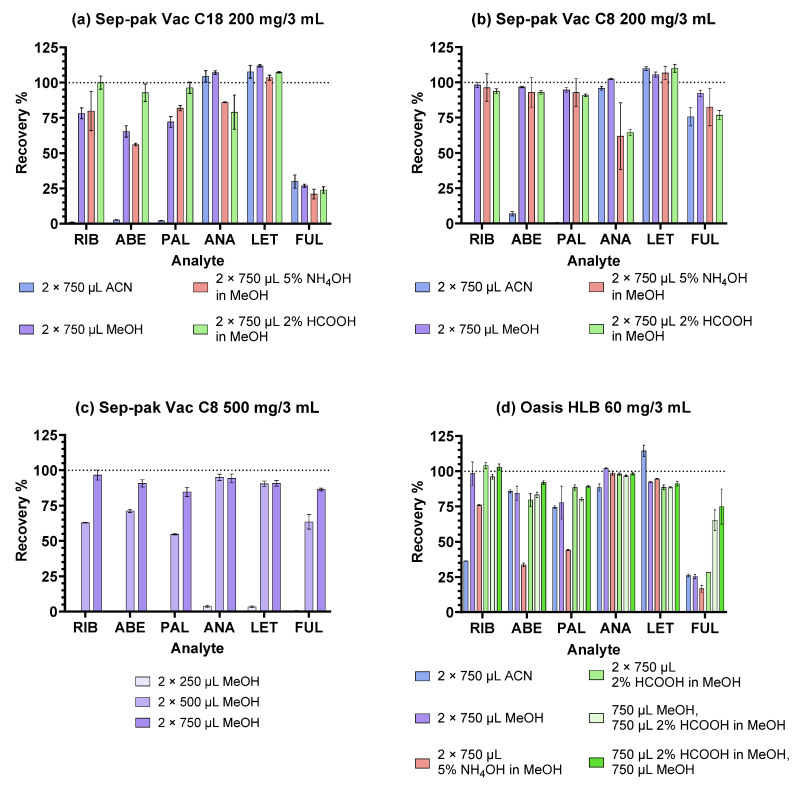
Extraction recoveries obtained with different eluents on reversed-phase sorbents.

The results are presented in Figure 1. As seen in Figure 1a, the highest recoveries on the highly hydrophobic C18 sorbent were obtained with MeOH and HCOOH in MeOH as elution solvents. This is also in accordance with our previous chromatographic experience: while using mobile phases without pH modifiers on a C18 column, increased retention of the weakly basic CDKi was observed, which was drastically reduced by the addition of HCOOH and their subsequent protonation. When comparing the SPE elution efficacy of ACN and MeOH, it can be concluded that the formation of hydrogen bonds with MeOH, a protic solvent, greatly increased analyte solubility, whereas the same effect could not be achieved with ACN, an aprotic solvent. Finally, the addition of ammonia to MeOH provided similar recoveries of the CDKi compared to MeOH alone since the degree of ionisation and, thus, the affinity of the CDKi for the sorbent was relatively similar, as seen from their pKa values (Table 1). A chromatogram of a blank plasma sample prepared using the C18 sorbent eluted with HCOOH in MeOH is shown in Supplementary Figure S1.

On the C8 sorbent, significantly higher recoveries of all the analytes for all tested conditions except for ACN were observed (Figure 1b). It is possible that FUL was too strongly retained in the structure of the C18 sorbent, whereas C8 offered a less lipophilic environment, which also proved favourable for the other analytes. The extracts obtained with MeOH as the eluent were both rich in the analytes of interest and free from the interfering matrix components (Supplementary Figure S2). 

Increasing volumes of MeOH were also tested on the C8 sorbent to assess if greater recoveries could be obtained. As discernible from Figure 1c, lower MeOH volumes were already sufficient for ANA and LET, while for the CDKi and FUL, maximal volumes were necessary to achieve the highest recoveries and precision. This indicates relatively stronger interactions of the CDKi and FUL with the sorbent. In the case of the CDKi, this is probably related to their low degree of ionisation at neutral pH, which causes their lower distribution into the elution solvent. Increasing the volume of the solvent increases the possibility of hydrogen bond formation, shifting this balance. For FUL, a strong interaction with the sorbent may be based on its steric properties—the long alkyl chain easily becomes trapped between the C8 chains of the sorbent; however, a large enough volume of the elution solvent is able to sufficiently solubilise the molecules. ANA and LET, on the other hand, are smaller compounds, less abundant in saturated alkyl groups, and are, therefore, likely to be more easily eluted from the sorbent, even with lower volumes.

In contrast to the silica-based C8 and C18 sorbents, Oasis HLB is a polymeric sorbent comprised of vinylpyrrolidone and divinylbenzene groups that enable both hydrophilic, dipole–dipole interactions and hydrophobic, especially π-π interactions, with the compounds from the sample [9]. It is to be expected that the analytes with more polar or aromatic groups will be more strongly retained. The results are shown in Figure 1d. Similar to the C8 and C18 sorbents, the CDKi are more easily solubilised and eluted when in their ionised forms or in a protic solvent. Consequently, their diminished recoveries were observed with ACN and NH_4_OH in MeOH, whereas solvent combinations including HCOOH in MeOH and/or only MeOH, proved favourable, which is also supported by the literature [24,25]. ANA and LET are eluted with similar efficiency, regardless of the elution solvent composition, since they are neutral in the whole studied pH range; therefore, their solubilisation is not dependent on their ionisation. For FUL, retention may be based on steric positioning, allowing for the π-π, hydrophobic, and dipole–dipole interactions to take place. Although HCOOH provided improved recoveries, especially for FUL, a significant amount of matrix interferences was visible in the chromatograms (Supplementary Figure S3); therefore, an SPE sorbent yielding a more selective extraction may be more adequate.

#### 2.1.2. Ion-Exchange Sorbents

As shown in Table 1, the CDKi are weak, basic compounds that predominantly exist in the cationic form below pH ≈ 9 [4]; therefore, different mixed-mode cation-exchange sorbents may be of interest. These sorbents are usually based on a polymeric backbone similar to HLB, thus offering additional retention mechanisms for ion exchange, which can benefit the non-ionised analytes, such as ANA, LET, and FUL. What is more, ANA and LET were successfully extracted using Strata X-C in the previously reported literature [23]. The structures of the ion-exchange sorbents used in this work are shown in Table 4 [7,28]. 

All tested conditions are listed in Table 5. To achieve analyte protonation, plasma samples were diluted with 2% H_3_PO_4_ and Milli-Q water at a ratio of 5:1:5 (experiments IV and V in Table 5) or with 100 mM sodium acetate (Na-Ac), pH 5.6, at a ratio of 4:5 (experiment VI) [29]. The washing step included flushing the sorbent with aqueous acid solutions, while MeOH with different pH modifiers was tested as the elution solvent.

**Figure 2:**
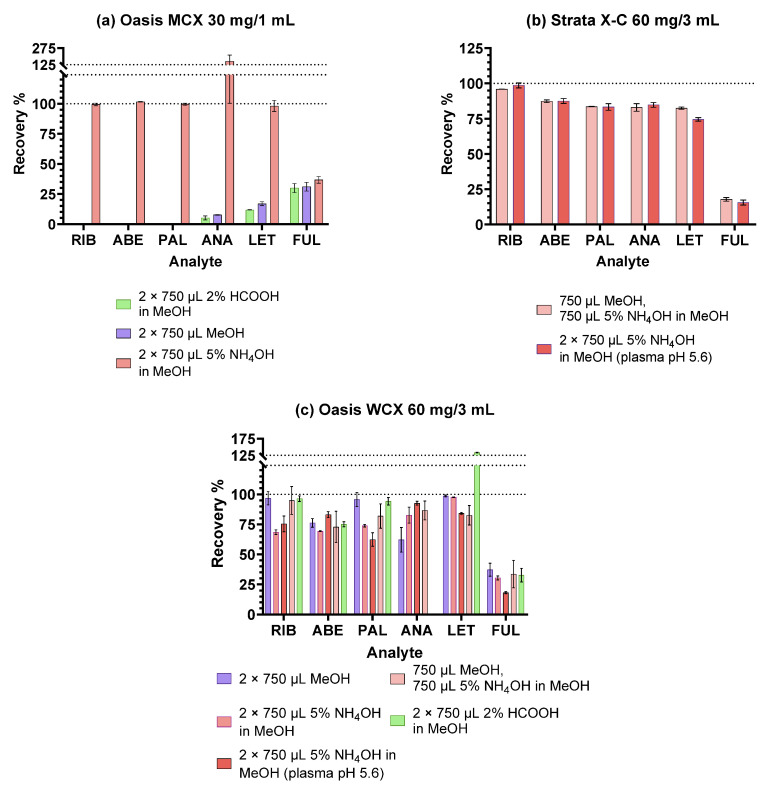
Extraction recoveries obtained with different eluents on ion-exchange sorbents.

The results are shown in Figure 2. Oasis MCX is a sorbent comprised of the same polymeric base as HLB, however, with the addition of sulfonic groups (pKa < 1) [8]. Thus, retention mechanisms of HLB are available together with the cation-exchanging properties. The retained cationic analytes can be eluted using an alkaline solution, shifting them to their non-ionic form [7]. The results, shown in Figure 2a, are in accordance with the expectations: no or very low recoveries of the CDKi and aromatase inhibitors were observed when applying neutral or acidic eluents, while high recoveries were obtained with NH_4_OH in MeOH. It is probable that the CDKi mostly entered electrostatic interactions with the sulfonic groups, while aromatase inhibitors and FUL also interacted with the divinylbenzene groups via the π-π and hydrophobic interactions. Chromatograms of the samples eluted with NH_4_OH in MeOH (Supplementary Figure S4) revealed some severe matrix interferences and low selectivity (also evident from the seemingly elevated recovery of ANA), which, along with the low recoveries of FUL, warrant the choice of different extraction conditions.

Strata X-C is another polymeric, mixed-mode cation-exchange sorbent for the extraction of weak bases, similar to the Oasis MCX; however, it was comprised only of divinylbenzene-sulfonic groups without the vinylpyrrolidone [8,30]. It is visible in Figure 2b that elution with NH_4_OH in MeOH or a two-step combination of MeOH and NH_4_OH in MeOH yielded practically equal results for each analyte and was slightly lower than with the Oasis MCX. It is not clear from the manufacturer data whether the degree of divinylbenzene sulfonation is higher than in the MCX, but it could, together with the lack of the vinylpyrrolidone groups, account for the observed lower recoveries. It can be assumed that ANA and LET could still be easily retained via interactions with divinylbenzene when unprotonated, due to their small molecular masses and significant number of π bonds, whereas the large and hydrophobic FUL could not. Judging by the structure of this sorbent (Table 4), a steric challenge to the binding of FUL may also be possible.

Oasis WCX is a polymer of vinylpyrrolidone and divinylbenzene, modified with carboxylic groups (pKa ≈ 5). It is meant for the extraction of stronger bases from the sample, assuming they remain protonated at higher pH values of the alkaline washing solvent. Ion exchange occurs when eluting with an acidic eluent and the protons from the solvent switch place with the analytes [7]. However, since none of the analytes of interest are strong bases, as shown in Table 1, the strong alkaline washing step proposed by the manufacturer was avoided in order to preserve their retention. The results obtained with this sorbent (Figure 2c) indicate the least precision of all the tested sorbents as well as the poorest cleanup. Variable matrix interferences coeluting with the analytes of interest were observed at all the tested conditions. Vague similarities with the results obtained with HLB can be observed—which is not surprising—since the same retention mechanism was most likely present for the analytes of interest. MeOH and HCOOH in MeOH provided slightly better overall recoveries than the NH_4_OH combinations; however, they showed a significant coelution of interferences with ANA and LET (evident from Supplementary Figures S5 and S6 and reflected in their recoveries). Since the full potential of the WCX sorbent could not be realised for these analytes, other sorbents may be more suitable.

**Figure 3:**
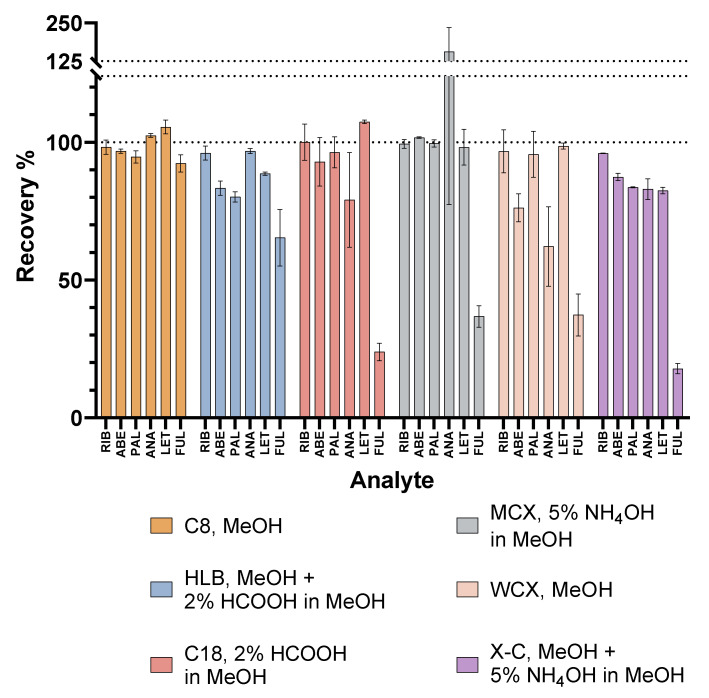
Cross-comparison of the best extraction recoveries with all the tested SPE sorbents.

To determine the best extraction conditions for all six analytes of interest, the most favourable conditions for each sorbent were mutually compared, as summarised in Figure 3.

As can be seen, the extraction recoveries of FUL were the poorest in most of the tested conditions, except for the C8 eluted with MeOH and the HLB eluted with a combination of MeOH and HCOOH in MeOH. For the other analytes, superior or similar extraction efficacies were achieved with the C8 eluted with MeOH and also with narrower error bars than in some other cases. The C18 sorbent eluted with HCOOH in MeOH and the X-C eluted with a combination of MeOH and NH_4_OH in MeOH also showed potential for the extraction of the combinations of only CDKi and aromatase inhibitors. However, SPE with a Sep-Pak Vac C8 column, 200 mg/3 mL, eluted with 2 × 750 µL MeOH, proved optimal for all six of the drugs of interest. This method was therefore transferred to LC-MS/MS, as described in Section 3.5., validated, and applied to real patient plasma samples. 

### 2.2. Method Validation

The following validation parameters were assessed for the newly developed SPE-LC-MS/MS method: linearity, calibration range, accuracy, precision, selectivity, carry-over, and matrix effects. The stability of the samples at the same storage and working conditions as used in this work has already been confirmed [12]. 

#### 2.2.1. Linearity and Calibration range

Fresh calibration curves were prepared each time analyte quantitation was planned. All calibration curves were weighted by 1/*x*^2^. At least 75% (six) of the calibration standard levels met the criteria that the accuracies of the back-calculated concentrations were within ±15% of the nominal values. The calibration results are summarised in Table 6. The extracted ion chromatograms (EIC) of all the analytes at the LLOQ concentration level are shown in Supplementary Figure S7.

#### 2.2.2. Accuracy and Precision

Accuracy and precision were assessed at three quality control (QC) concentration levels across the linear range: lower limit of quantitation (LLOQ), low, and high, on 10 samples per concentration level within one day and a total of 15 samples per concentration level between days. They were found to be acceptable at all concentration levels (the bias was within ±15%, and the RSD was below 15%). The results are shown in Table 7. All QCs were quantitated according to a fresh calibration curve. 

#### 2.2.3. Selectivity and Carry-Over 

Selectivity was assessed during the method development by reviewing chromatograms of blank plasma samples. The blank chromatogram obtained with the optimised C8-SPE method (Supplementary Figure S2) showed no significant interferences at the retention times of the analytes.

The presence of carry-over was checked by injecting blank samples after the highest calibration concentrations. A slight carry-over of RIB and PAL was observed. An additional blank injection was introduced for the needle and column cleanup, and the needle was washed for 100 s with 50% MeOH after each injection. Thus, the carry-over of RIB and PAL was reduced to less than 3% of the LLOQ (Supplementary Figure S8).

#### 2.2.4. Matrix Effects

The matrix effects were tested at two QC concentration levels (low and high) in triplicate on plasma samples from six different individuals, including a haemolysed and lipemic plasma, and calculated according to Equation (1):

(1) $$\mathrm{Matrix}\;\mathrm{effect}\;\left(\%\right)=\frac{\mathrm{Signal}\;\mathrm{in}\;\mathrm{the}\;\text{post-extraction}\;\mathrm{spiked}\;\mathrm{sample}}{\mathrm{Signal}\;\mathrm{in}\;\mathrm{the}\;\mathrm{standard}\;\mathrm{solution}}*100\%-100\%$$

**Figure 4:**
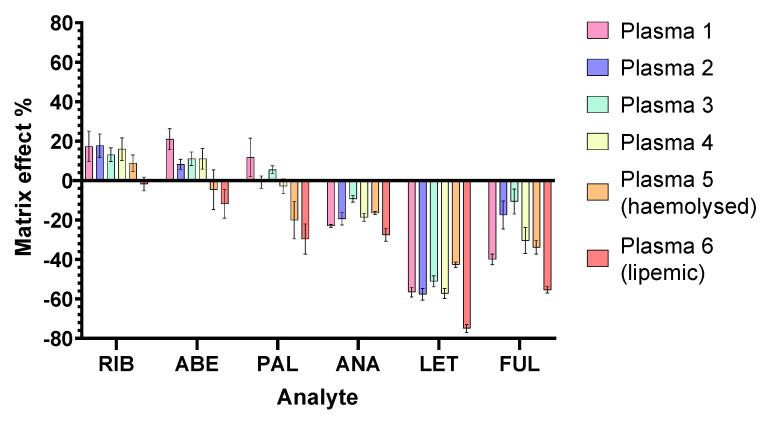
Matrix effects: mean and SD of all the collected data from six different lots; n = 6 samples per plasma lot.

The results are depicted in Figure 4 and Supplementary Table S1. Differences in the matrix effects between the low and high QC concentrations were negligible. It is discernible that stronger ion suppression was present in the cases of LET and FUL. All analytes exhibited ion suppression in lipemic plasma, with some ion enhancement otherwise present for the CDKi. The variability between different sources of plasma was most pronounced for FUL (peak area RSD 25.21%) and the least for RIB (peak area RSD 5.16%). Evidently, although simultaneous extraction of all the analytes, as well as the cleanup of most of the UV-absorbing interferences, was achieved, plasma lipid removal was not as thorough.

It should be noted that extensive signal instability was also observed in the case of FUL, which sometimes manifested in a nonlinear response. This phenomenon is attributed to the drug’s high lipophilicity, low ionisation efficiency in the positive ESI mode, and high susceptibility to ionisation effects from any leftover plasma interferences accumulated at the LC-MS interface. Regular, thorough rinsing of the spray shield with 50% i-propanol alleviated the issue to a certain extent; however, the introduction of an isotopically labelled analogue as the internal standard is highly recommended in the case of routine applications in clinical practice. In this work, the main goal was to achieve successful simultaneous extraction of the analytes from plasma samples using SPE and identify potential pitfalls for the method’s application. Further addressing the MS issues and routine application of the method are outside the scope of this paper but will be the subject of our future efforts.

### 2.3. Analysis of Patient Samples

To prove the applicability of the method on real patient samples, plasma from four breast cancer patients treated with CDKi + ET combinations was prepared using the developed procedure and quantitated according to a fresh calibration curve. Each of the analytes was present in at least one of the patients. The results were as follows: patient 1: RIB 981.7 ng/mL, ANA 41.7 ng/mL; patient 2: ABE 276.4 ng/mL, LET 69.8 ng/mL; patient 3: PAL 74.2 ng/mL, FUL 21.0 ng/mL; patient 4: PAL 119.3 ng/mL, FUL 14.2 ng/mL. All determined concentrations are within the method’s validated linear ranges as well as within the expected concentration ranges of these drugs in patient plasma [31,32,33,34,35,36], which proves the feasibility of the proposed extraction method. Chromatograms of the patient samples are shown in Supplementary Figure S9.

### 2.4. Comparison with Previously Published Methods

Key parameters of the proposed method and the relevant previously published works are shown in Table 8. The procedure developed in this work is the first reported SPE method for the simultaneous extraction of all six breast cancer drugs of interest from plasma samples. In regard to the SPE methods for any of these analytes [14,23,24,25], novel SPE conditions were optimised. Plasma samples were diluted with Milli-Q water and applied onto a C8 SPE column after sorbent conditioning with MeOH and water. The sorbent was washed with water and 5% MeOH, thus removing the plasma proteins, and the analytes were eluted with two portions of MeOH. The obtained extraction recoveries for all the analytes were higher than in any of the previously published works. The method’s linear ranges are clinically relevant, according to the expected patient plasma concentrations, and were found suitable in the conducted patient samples’ analysis. Adequate precision and accuracy of the method in the upper and lower determination ranges have been confirmed.

In relation to other methods for these six breast cancer drugs, where samples are prepared using less selective PPT or labour-intensive DLLME [5,11,12], the proposed method explores a novel sample preparation approach. SPE is simple and selective when fully developed, and this work offers insight into the potential advantages as well as drawbacks of certain commercially available sorbents in the described application. The newly developed and validated C8-SPE method shows excellent extraction recoveries and sample cleanup, offering a novel, automatable approach to clinical application for the drugs of interest. What is more, since different interaction mechanisms are explored in depth in this article, the method development procedure may also be helpful for other structurally related compounds. The silica-based C8 sorbent itself is proven a versatile and efficient tool, retaining its applicability even beside the new-generation polymeric sorbents.

## 3. Materials and Methods

### 3.1. Chemicals and Reagents

MeOH for the HPLC and MS, as well as ACN for the HPLC, were purchased from J.T. Baker (Phillipsburg, NJ, USA); HCOOH for the LC-MS was purchased from Supelco (St. Louis, MO, USA); H_3_PO_4_ (85%) was purchased from T.T.T. (Sveta Nedelja, Croatia); NH_4_OH (25%) and Na-Ac were from Alkaloid (Skopje, North Macedonia); and HCl (37%) was from VWR (Radnor, PA, USA). Ultrapure water was obtained using a Merck Millipore Milli-Q IQ 7015 system (Darmstadt, Germany). Analytical grade standards (purity > 97%) of PAL, RIB, and ABE were from Toronto Research Chemicals (Toronto, Canada); ANA and LET were from Tokyo Chemical Industry (Tokyo, Japan); and FUL was from MilliporeSigma (Burlington, MA, USA).

### 3.2. Preparation of the Standard Solutions

The primary stock solutions of RIB, ABE, ANA, LET, and FUL were prepared to the concentration of 1 mg/mL in MeOH. The primary stock solution of PAL was prepared to the concentration of 225 µg/mL in H_2_O:ACN 50:50 *v*/*v*. These solutions were mixed and diluted in MeOH to obtain working solutions of the appropriate concentrations. The concentrations of the calibrants used in the method validation are shown in Table 9. All the solutions were kept refrigerated at 4 °C and were stable for at least three months.

### 3.3. Plasma Sample Pretreatment

Blood from the patients treated with the drugs of interest, as well as drug-free blood from healthy volunteers, was collected in vials with the K_2_-EDTA anticoagulant. After centrifugation at 1500× *g* for 10 min, the supernatant was stored short-term at −18 °C and long-term at −80 °C. The plasma was thawed at room temperature for 30 min before any experiments were conducted. Research approvals were obtained from the Ethics Committee of the University of Zagreb Faculty of Pharmacy and Biochemistry (approval 251-62-03-19-30) and the Ethics Committee of University Hospital Centre Zagreb (approval 02/21-JG).

### 3.4. Plasma Sample Preparation

The tested extraction phases, Oasis MCX 30 mg/1 mL, Oasis HLB 60 mg/3 mL, Oasis WCX 60 mg/3 mL, Sep-Pak Vac C18 200 mg/3 mL, Sep-Pak Vac C8 200 mg/3 mL, and Sep-Pak Vac C8 500 mg/3 mL, were obtained from Waters (Milford, MA, USA), while the Strata-X-C 60 mg/3 mL was from Phenomenex (Torrance, CA, USA). 

Before commencing with the SPE procedure, blank plasma samples were spiked with a working analyte standard solution at the ratio of plasma:standard 9:1 *v*/*v* and the samples were diluted with an appropriate solvent. The extractions were carried out using a vacuum manifold (Supelco, Bellefonte, PA, USA) equipped with a vacuum pump. A known volume of the sample eluate (80% of the nominal eluate volume) was evaporated to dryness on a vacuum concentrator (Eppendorf, Hamburg, Germany) and reconstituted in 80 µL of 65% MeOH. Once prepared, the samples were kept on the autosampler at 10 °C for no longer than 10 h.

### 3.5. Chromatographic Conditions

A Waters XBridge BEH phenyl column, with the dimensions of 150 × 4.6 mm and particle size 2.5 µm, with a corresponding VanGuard phenyl guard column (Waters, Milford, MA, USA), was used as the stationary phase, thermostated at 35 °C. Ultrapure water (phase A) and MeOH (phase B), both containing 0.1% HCOOH, were used as the mobile phase in the gradient elution. The initial experiments were performed on an Agilent 1100 HPLC equipped with DAD and FLD (Agilent Technologies, Santa Clara, CA, USA). The flow rate was set to 0.8 mL/min, and 10 µL of the sample was injected. The applied mobile phase gradient is shown in Table 10.

The method was then transferred to an Agilent 1290 Infinity II UHPLC coupled to a 6470 triple-quadrupole mass spectrometer (QQQ-MS) equipped with an Agilent Jet Stream electrospray (AJS-ESI) source (Agilent Technologies, Santa Clara, CA, USA) to achieve the necessary detection sensitivity. With the same stationary and mobile phases, the flow rate was adjusted to 0.6 mL/min using the gradient elution described in Table 11. An adjusted gradient composition was necessary due to the smaller dwell volume of the UHPLC system. The column temperature was 35 °C, and 10 µL of the sample was injected into the system.

### 3.6. Detection Conditions

**Figure 5:**
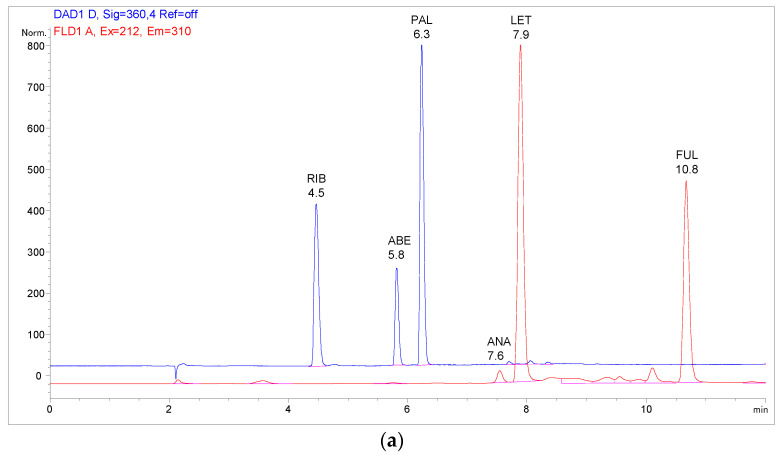

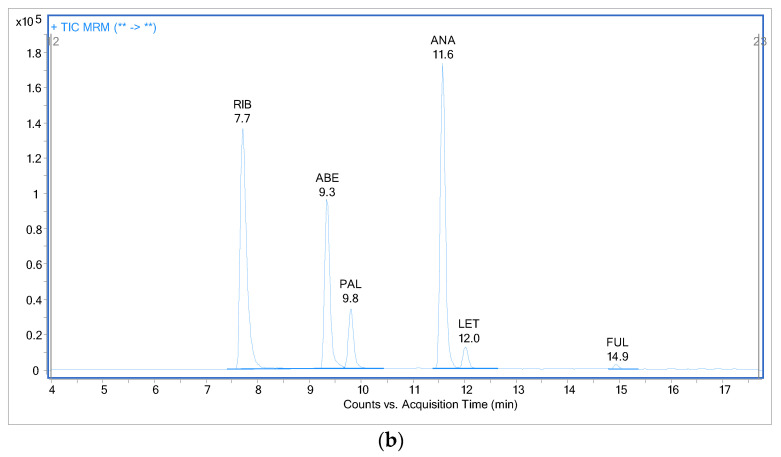
Representative chromatograms of a spiked plasma sample prepared using the optimal C8-SPE sample preparation method: (**a**) HPLC-DAD-FLD: DAD 360 nm (blue), FLD Ex 212, Em 310 nm (red), concentration of all analytes 10 µg/mL; (**b**) UHPLC-MS/MS: total ion chromatogram (TIC) at the LLOQ concentration level.

In the preliminary studies, the analytes were detected using DAD on 360 nm for PAL, RIB, and ABE, together with FLD at a 212 nm excitation (Ex) and a 310 nm emission (Em) for ANA, LET, and FUL, as previously reported [5]. The AJS-ESI-MS conditions were as follows: gas temperature 300 °C, gas flow 13 L/min, nebulizer pressure 35 psi, sheath gas temperature 350 °C, sheath gas flow 12 L/min, positive ESI mode with capillary voltage 3500 V, nozzle voltage 500 V, and cell accelerator voltage 5 V. Analyte-specific parameters of the monitored mass transitions are shown in Table 12, while the representative chromatograms, obtained using both LC methods, are shown in Figure 5. Exemplary mass spectra of all the analytes are provided in Supplementary Figure S10.

### 3.7. Data Collection and Analysis

Data were collected using the Agilent Chemstation 10 software (Santa Clara, CA, USA) for the Agilent 1100 HPLC, and the Agilent MassHunter Workstation 10.0 software was used for the data acquisition and qualitative analysis (Santa Clara, CA, USA) for the Agilent 1290 UHPLC. Data analyses were performed using Microsoft 365 Excel (Redmond, WA, USA) and GraphPad Prism 8 (Boston, MA, USA).

## 4. Conclusions

In recent years, the importance of TDM of anticancer drugs has been an emerging topic. An increasing number of bioanalytical methods are being reported in search of fulfilling the first precondition for TDM testing: the development of a reliable procedure for the quantitative determination of drugs-candidates in biological matrices suitable for the busy clinical setting. SPE is currently one of the most common sample preparation techniques due to its versatility. It is simple and fast once optimised; however, its implementation requires extensive method development. In this work, different extraction conditions were tested (HLB, reversed-phase C8 and C18, and ion-exchange sorbents WCX, MCX, and X-C, combined with various elution solvents) for the extraction of six breast cancer drugs, palbociclib, ribociclib, abemaciclib, anastrozole, letrozole, and fulvestrant, from human plasma samples.

Of all the tested conditions, the Sep-Pak Vac C8 (200 mg/3 mL) sorbent eluted with MeOH shows the best overall extraction yields and cleanup for all six drugs of interest, while the Sep-Pak Vac C18 (200 mg/3 mL) eluted with HCOOH in MeOH and the Strata X-C (60 mg/3 mL) eluted with a combination of MeOH and NH_4_OH in MeOH show potential for combinations of only CDKi and aromatase inhibitors. These findings, backed by the detailed analysis of the potential interaction mechanisms, may also be a useful starting point for the development of SPE methods for other structurally similar drugs of interest.

The proposed C8-SPE-LC-MS/MS method is simple and fast, ecologically acceptable, precise, accurate, and sensitive, with adequate, clinically relevant linear ranges. Potential pitfalls in the method development and its application are acknowledged, such as the high levels of interference, regardless of the acceptable extraction yields in the case of the MCX and WCX sorbents, or the observed matrix effects with the C8 sorbent for some of the analytes. This can be avoided in routine clinical settings with regular and thorough ion source rinsing and by using isotopically labelled internal standards. Carry-over was observed during the method development and was resolved with additional column rinsing. Nevertheless, the method’s feasibility is established by its application on four samples from patients treated with the drugs of interest. To the best of our knowledge, this is the first SPE method for the simultaneous extraction of ABE, RIB, PAL, ANA, LET, and FUL from plasma samples, demonstrating satisfactory sensitivity, selectivity, and precision, together with exceedingly high recoveries (≥ 92.3%). 

## Figures and Tables

**Table 1:** Physical-chemical properties and structural formulae of the drugs of interest with highlighted pKa values (corresponding to the proton denoted in red), and functional groups for potential intermolecular interactions: π-π interactions (purple), hydrophobic interactions (green), and hydrogen bonds (blue).

Analyte	RIB	PAL
**Structure, interactions, pKa**	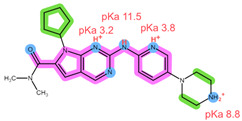	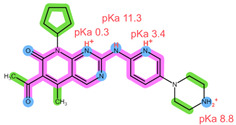
**Emp. formula** **Molar mass** **logP** **HB donors** **HB acceptors** **TPSA** **Rotatable bonds**	C_23_H_30_N_8_O 434.5 g/mol 2.04 2 5 91.21 Å² 5	C_24_H_29_N_7_O_2_ 447.5 g/mol 2.39 2 6 105.04 Å² 5
**Analyte**	**ABE**	**FUL**
**Structure, interactions, pKa**	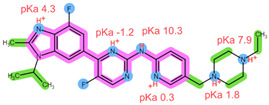	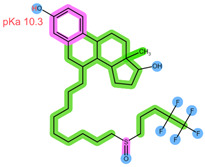
**Emp. formula** **Molar mass** **logP** **HB donors** **HB acceptors** **TPSA** **Rotatable bonds**	C_27_H_32_F_2_N_8_ 506.6 g/mol 4.04 1 8 75.00 Å² 7	C_32_H_47_F_5_O_3_S 606.8 g/mol 8.06 2 8 76.74 Å² 14
**Analyte**	**ANA**	**LET**
**Structure, interactions, pKa**	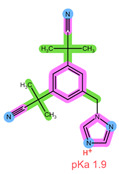	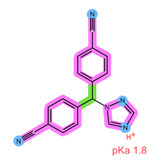
**Emp. formula** **Molar mass** **logP** **HB donors** **HB acceptors** **TPSA** **Rotatable bonds**	C_17_H_19_N_5_ 293.4 g/mol 2.35 0 4 78.29 Å² 4	C_17_H_11_N_5_ 285.3 g/mol 2.32 0 4 78.29 Å² 3

Abbreviations: Emp.: empirical; HB: hydrogen bond; TPSA: total polar surface area.

**Table 2:** Structural formulae of the tested reversed-phase sorbents with highlighted functional groups and pKa for potential intermolecular interactions: π-π interactions (purple), hydrophobic interactions (green), hydrogen bonds (blue), and dipole-based interactions (yellow).

Sorbent	Sep-Pak Vac C18	Sep-Pak Vac C8	Oasis HLB
Structure	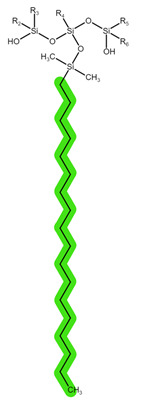	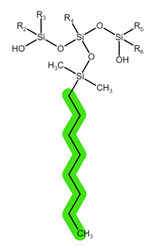	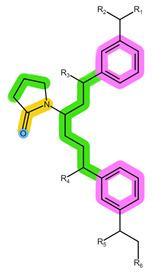

**Table 3:** The SPE procedures used for the reversed-phase sorbents.

Experiment	I.	II.
**SPE cartridges**	Oasis HLB 60 mg/3 mL Sep-Pak Vac C18 200 mg/3 mL Sep-Pak Vac C8 200 mg/3 mL	Oasis HLB 60 mg/3 mL Sep-Pak Vac C8 500 mg/3 mL
**1. Conditioning**	2 mL MeOH 2 mL H_2_O	2 mL MeOH 2 mL H_2_O
**2. Sample addition (400 µL diluted plasma sample)**		
**3. Washing**	1 mL H_2_O 1 mL 5% MeOH	1 mL H_2_O 1 mL 5% MeOH
**4. Elution**	2 × 750 µL MeOH or 2 × 750 µL ACN or 2 × 750 µL 5% NH_4_OH in MeOH or 2 × 750 µL 2% HCOOH in MeOH	**HLB:** 750 µL MeOH and 750 µL 2% HCOOH in MeOH or 750 µL 2% HCOOH in MeOH and 750 µL MeOH **C8:** 2 × 250 µL MeOH or 2 × 500 µL MeOH or 2 × 750 µL MeOH

**Table 4:** Structural formulae of the tested ion-exchange sorbents with highlighted functional groups and pKa for potential intermolecular interactions: π-π interactions (purple), hydrophobic interactions (green), hydrogen bonds (blue), electrostatic interactions (red), and dipole-based interactions (yellow).

Sorbent	Oasis MCX	Oasis WCX	Strata X-C
Structure	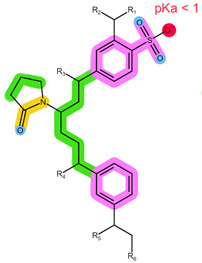	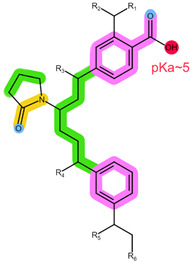	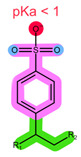

**Table 5:** SPE procedures used for the ion-exchange sorbents.

Experiment	IV.	V.	VI.
**SPE cartridges**	Oasis MCX 30 mg/1 mL Oasis WCX 60 mg/3 mL	Oasis WCX 60 mg/3 mL Strata-X-C 60 mg/3 mL	Oasis WCX 60 mg/3 mL Strata-X-C 60 mg/3 mL
**1. Conditioning**	2 mL MeOH 2 mL 0.2% H_3_PO_4_	2 mL MeOH 2 mL 0.2% H_3_PO_4_	1 mL MeOH 1 mL 100 mM Na-Ac (pH 5.6)
**2. Sample addition (400 µL diluted plasma sample)**			
**3. Washing**	3 mL 0.2% H_3_PO_4_ 2 mL 0.1 M HCl	3 mL 0.2% H_3_PO_4_ 2 mL 0.1 M HCl	1 mL Na-Ac (pH 5.6) 1 mL MeOH:100 mM Na-Ac (pH 5.6) = 2:8
**4. Elution**	2 × 750 µL MeOH or 2 × 750 µL 5% NH_4_OH in MeOH or 2 × 750 µL 2% HCOOH in MeOH	750 µL MeOH and 750 µL 5% NH_4_OH in MeOH	2 × 750 µL 5% NH_4_OH in MeOH

**Table 6:** Calibration results.

Analyte	RIB	ABE	PAL	ANA	LET	FUL
**Range (ng/mL)**	700–3500	80–400	40–200	20–100	40–200	10–50
**Slope**	1103	10,846	11,875	68,230	5066	3013
**Intercept**	620,726	613,810	−6744	−28,605	4075	−17,390
**R**	0.9953	0.9933	0.9970	0.9983	0.9948	0.9972
**N(points)**	7	7	7	7	7	7
Max. %bias *	5.77	−7.85	4.62	−5.01	8.06	−6.60

* The accuracy of the back-calculated concentrations of each calibration standard.

**Table 7:** Results of accuracy and precision tests.

Analyte	Concentration (ng/mL)	Intra-Day, n = 10		Inter-Day, n = 15	
		Bias%	RSD%	Bias%	RSD%
**RIB**	700	0.6	7.1	1.3	6.6
	1120	9.5	1.8	10.4	2.9
	2800	−3.2	1.8	−2.8	2.0
**ABE**	80	−12.1	12.2	−7.7	11.3
	128	−1.3	3.9	4.2	6.1
	320	−2.3	3.1	−3.6	3.0
**PAL**	40	7.0	9.1	4.7	8.2
	64	10.0	4.7	8.1	4.3
	160	3.1	3.0	−0.5	4.9
**ANA**	20	−6.0	9.6	−2.0	8.8
	32	−4.3	6.6	−0.4	7.3
	80	−5.8	4.4	1.1	8.6
**LET**	40	7.3	9.2	3.2	9.0
	64	6.0	8.6	4.6	9.1
	160	−3.9	12.4	1.4	13.4
**FUL**	10	8.2	14.2	13.5	14.3
	16	8.7	14.8	2.0	14.3
	40	−11.5	6.6	−12.7	12.9

**Table 8:** Comparison of the developed SPE-LC-MS/MS method with previously published methods.

Reference	Analytes	Analytical Technique	Sample Preparation Technique	Mean Extraction Recovery	Linear Range
Beer et al., 2010. [23]	ANA, LET, tamoxifen	LC-MS/MS	SPE w/Strata X-C (200 mg/3 mL)	92% ANA, 89% LET	5–200 ng/mL ANA, 10–300 ng/mL LET
Chavan et al., 2018. [14]	PAL	LC-MS/MS	PPT w/ACN, evaporation, SPE w/Phenomenex C18	n.a.	n.a.
Nalanda et al., 2018. [24]	PAL	LC-DAD	SPE w/Oasis HLB (30 mg/1 mL)	72.1%	100–3000 ng/mL
Leenhardt et al., 2021. [25]	PAL, RIB	LC-MS/MS	SPE w/Oasis HLB (30 mg/1 mL)	n.a.	3.9–129 ng/mL
Sato et al., 2021. [11]	PAL, RIB, ABE, ANA, LET, FUL	LC-MS/MS	PPT w/ACN:MeOH 9:1	n.a.	1–75 ng/mL ANA and FUL, 5–375 ng/mL PAL, 10–750 ng/mL ABE and LET, 100–7500 ng/mL RIB
Turković et al., 2022. [12]	PAL, RIB, ABE, ANA, LET, FUL	LC-MS/MS	PPT w/ACN	>85%	1–200 ng/mL ANA, 2.5–500 ng/mL LET, 3.1–500 ng/mL PAL, 5–1000 ng/mL FUL, 15–3000 ng/mL ABE, 25–5000 ng/mL RIB
Turković et al., 2023. [5]	PAL, RIB, ABE, ANA, LET, FUL	LC-DAD- FLD	PPT w/ACN, DLLME	81.7–95.6%	2.50–60.0 µg/mL ANA, 0.04–1.00 µg/mL LET, 0.08–1.92 µg/mL PAL, 0.50–12.0 µg/mL FUL, 0.11–2.61 µg/mL ABE, 0.25–5.95 µg/mL RIB
This work	PAL, RIB, ABE, ANA, LET, FUL	LC-MS/MS	SPE w/Sep-Pak Vac C8 (200 mg/ 3 mL)	92.3–105.5%	20–100 ng/mL ANA, 40–200 ng/mL LET and PAL, 10–50 ng/mL FUL, 80–400 ng/mL ABE, 700–3500 ng/mL RIB

w/: with; n.a.: not available.

**Table 9:** Concentration levels of the calibrant plasma samples (ng/mL).

Calibrant	RIB	ABE	PAL	ANA	LET	FUL
1	700	80	40	20	40	10
2	1120	128	64	32	64	16
3	1400	160	80	40	80	20
4	1750	200	100	50	100	25
5	2100	240	120	60	120	30
6	2520	288	144	72	144	36
7	2800	320	160	80	160	40
8	3500	400	200	100	200	50

**Table 10:** Mobile phase gradient applied on the Agilent 1100 HPLC.

Time (min)	Mobile Phase B (%)
0.0	30.0
5.5	85.0
9.5	90.0
10.0	100.0
16.5	100.0
17.0	30.0
30.0	30.0

**Table 11:** Mobile phase gradient applied on the Agilent 1290 UHPLC.

Time (min)	Mobile Phase B (%)
0.0	5.0
10.5	85.0
14.5	90.0
15.0	100.0
20.0	100.0
20.5	30.0
26.0	30.0

**Table 12:** Optimised MS/MS transitions for each analyte.

Analyte	Precursor Ion (*m*/*z*)	Product Ion (*m*/*z*)	CE (V)	Dwell Time (ms)	Fragmentor (V)
**RIB**	435.2	322.1	25	200	200
		252.1	30		
**ABE**	507.2	393.1	25	60	140
		245.0	75		
**PAL**	448.0	380.3	30	60	180
		362.0	45		
**ANA**	294.1	225.4	25	60	140
		115.2	70		
**LET**	217.0	190.3	25	60	120
		164.1	50		
**FUL**	607.4	589.0	15	200	160
		467.0	29		

CE: collision energy.

## Data Availability

Data are contained within the article and Supplementary Material.

## Supplementary Materials

The following supporting information can be downloaded at: https://www.mdpi.com/article/10.3390/ph16101445/s1, Figure S1: Chromatogram of a blank plasma sample prepared using the Sep-Pak Vac C18 sorbent (200 mg/3 mL) eluted with HCOOH in MeOH. Detection wavelengths: DAD 360 nm (blue), FLD Ex 212, Em 310 nm (red); Figure S2: Chromatogram of a blank plasma sample prepared using the Sep-Pak Vac C8 sorbent (200 mg/3 mL) eluted with MeOH. Detection wavelengths: DAD 360 nm (blue) FLD Ex 212, Em 310 nm (red); Figure S3: Chromatogram of a blank plasma sample prepared using the Oasis HLB sorbent (60 mg/3 mL) eluted with HCOOH in MeOH. Detection wavelengths: DAD 360 nm (blue) FLD Ex 212, Em 310 nm (red); Figure S4: Chromatogram of a blank plasma sample prepared using the Oasis MCX sorbent (30 mg/1 mL) eluted with 5% NH_4_OH in MeOH. Detection wavelengths: DAD 360 nm (blue) FLD Ex 212, Em 310 nm (red); Figure S5: Chromatogram of a blank plasma sample prepared using the Oasis WCX sorbent (60 mg/3 mL) eluted with MeOH. Detection wavelengths: DAD 360 nm (blue) FLD Ex 212, Em 310 nm (red); Figure S6: Chromatogram of a blank plasma sample prepared using the Oasis WCX sorbent (60 mg/3 mL) eluted with HCOOH in MeOH. Detection wavelengths: DAD 360 nm (blue) FLD Ex 212, Em 310 nm (red); Figure S7: Extracted ion chromatograms of the quantifier ion transition for the analytes at the LLOQ concentration level: a) RIB, b) ABE, c) PAL, d) ANA, e) LET, and f) FUL; Figure S8: Overlaid extracted ion chromatograms of the quantifying ion transitions for all analytes at the LLOQ concentration levels and in a blank sample injected after the ULOQ concentration level. Brown: RIB; dark blue: ABE; dark red: PAL; light green: ANA; light blue: LET; dark green: FUL; Figure S9: MS chromatograms of the samples from a) patient 1 (RIB, ANA); b) patient 2 (ABE, LET); c) patient 3 (PAL, FUL); and d) patient 4 (PAL, FUL); Figure S10: Exemplary MS spectra of the analytes: a) RIB (precursor ion *m*/*z* 435.2; CE 40 V, fragmentor 200 V), b) ABE (precursor ion *m*/*z* 507.2; CE 30 V, fragmentor 140 V), c) PAL (precursor ion *m*/*z* 448.0; CE 40 V, fragmentor 180 V), d) ANA (precursor ion *m*/*z* 294.1; CE 30 V, fragmentor 140 V), e) LET (precursor ion *m*/*z* 217.0; CE 50 V, fragmentor 120 V), and f) FUL (precursor ion *m*/*z* 607.4; CE 30 V, fragmentor 160 V); Table S1: Overall matrix effects results—average value of matrix effects and peak area RSD between all tested samples for each analyte.

## References

[1] Engler T., Fasching P.A., Lüftner D., Hartkopf A.D., Müller V., Kolberg H.C., Hadji P., Tesch H., Häberle L., Ettl J. (2022). Implementation of CDK4/6 Inhibitors and its Influence on the Treatment Landscape of Advanced Breast Cancer Patients—Data from the Real-World Registry PRAEGNANT. Geburtshilfe Frauenheilkd..

[2] Mueller-Schoell A., Groenland S.L., Scherf-Clavel O., van Dyk M., Huisinga W., Michelet R., Jaehde U., Steeghs N., Huitema A.D.R., Kloft C. (2021). Therapeutic drug monitoring of oral targeted antineoplastic drugs. Eur. J. Clin. Pharmacol..

[3] Pena-Pereira F., Wojnowski W., Tobiszewski M. (2020). AGREE—Analytical GREEnness Metric Approach and Software. Anal. Chem..

[4] Mlinarić Z., Turković L., Begović I., Nigović B., Sertić M. (2022). Rapid Capillary Electrophoresis Method for Simultaneous Determination of Abemaciclib, Ribociclib, and Palbociclib in Pharmaceutical Dosage Forms: A Green Approach. Molecules.

[5] Turković L., Koraj N., Mlinarić Z., Silovski T., Crnković S., Sertić M. (2023). Optimisation of dispersive liquid-liquid microextraction for plasma sample preparation in bioanalysis of CDK4/6 inhibitors in therapeutic combinations for breast cancer treatment. Heliyon.

[6] Sentellas S., Saurina J., Núñez O., Poole C.F. (2020). Solid-phase extraction in bioanalytical applications. Solid-Phase Extraction.

[7] Waters Oasis Sample Extraction Products—Taking the Complexity Out of SPE Method Development. https://www.waters.com/webassets/cms/library/docs/720005685en.pdf.

[8] Fontanals N., Marcé R.M., Borrull F., Cormack P.A.G. (2010). Mixed-mode ion-exchange polymeric sorbents: Dual-phase materials that improve selectivity and capacity. TrAC Trends Anal. Chem..

[9] Dias N.C., Poole C.F. (2002). Mechanistic study of the sorption properties of Oasis^®^ HLB and its use in solid-phase extraction. Chromatographia.

[10] Mutavdžić Pavlović D., Babić S., Horvat A.J.M., Kaštelan-Macan M. (2007). Sample preparation in analysis of pharmaceuticals. TrAC Trends Anal. Chem..

[11] Sato Y., Shigeta K., Hirasawa T., Sato T., Ogura J., Maekawa M., Ebata A., Hamanaka Y., Tada H., Ishida T. (2021). Establishment of an analytical method for simultaneous quantitation of CDK4/6 inhibitors, aromatase inhibitors, and an estrogen receptor antagonist in human plasma using LC-ESI-MS/MS. J. Chromatogr. B Anal. Technol. Biomed. Life Sci..

[12] Turković L., Bočkor L., Ekpenyong O., Silovski T., Lovrić M., Crnković S., Nigović B., Sertić M. (2022). Development and Validation of a Novel LC-MS/MS Method for the Simultaneous Determination of Abemaciclib, Palbociclib, Ribociclib, Anastrozole, Letrozole, and Fulvestrant in Plasma Samples: A Prerequisite for Personalized Breast Cancer Treatment. Pharmaceuticals.

[13] Gumustas M., Sengel-Turk C.T., Hascicek C., Ozkan S.A. (2014). Optimization of a validated stability-indicating RP-LC method for the determination of fulvestrant from polymeric based nanoparticle systems, drugs and biological samples. Biomed. Chromatogr..

[14] Chavan B.B., Tiwari S., Shankar G., Nimbalkar R.D., Garg P., Srinivas R., Talluri M.V.N.K. (2018). In vitro and in vivo metabolic investigation of the Palbociclib by UHPLC-Q-TOF/MS/MS and in silico toxicity studies of its metabolites. J. Pharm. Biomed. Anal..

[15] Martínez-Chávez A., Rosing H., Hillebrand M., Tibben M., Schinkel A.H., Beijnen J.H. (2019). Development and validation of a bioanalytical method for the quantification of the CDK4/6 inhibitors abemaciclib, palbociclib, and ribociclib in human and mouse matrices using liquid chromatography-tandem mass spectrometry. Anal. Bioanal. Chem..

[16] Soledad Poetto A., Posocco B., Zanchetta M., Gagno S., Orleni M., Canil G., Alberti M., Puglisi F., Toffoli G. (2022). A new LC-MS/MS method for the simultaneous quantification of abemaciclib, its main active metabolites M2 and M20, and letrozole for therapeutic drug monitoring. J. Chromatogr. B Anal. Technol. Biomed. Life Sci..

[17] Posocco B., Buzzo M., Poetto A.S., Orleni M., Gagno S., Zanchetta M., Iacuzzi V., Guardascione M., Puglisi F., Basile D. (2020). Simultaneous quantification of palbociclib, ribociclib and letrozole in human plasma by a new LC-MS/MS method for clinical application. PLoS ONE.

[18] Habler K., Kalla A.S., Rychlik M., Vogeser M., Teupser D. (2023). Therapeutic drug monitoring in breast cancer therapy—LC-MS/MS method for quantification of the CDK4/6 inhibitors abemaciclib, palbociclib, ribociclib, and major metabolites abemaciclib M20 and M2 in human serum. J. Pharm. Biomed. Anal..

[19] Margaryan T., Elliott M., Sanai N., Tovmasyan A. (2022). Simultaneous determination of LY3214996, abemaciclib, and M2 and M20 metabolites in human plasma, cerebrospinal fluid, and brain tumor by LC-MS/MS. J. Pharm. Anal..

[20] Burke S.M., Kamal M.K., Goey A.K. (2023). Development and Validation of a Quantitative LC-MS/MS Method for CDK4/6 Inhibitors Palbociclib, Ribociclib, Abemaciclib, and Abemaciclib-M2 in Human Plasma. Ther. Drug Monit..

[21] Alegete P., Kancherla P., Albaseer S.S., Boodida S. (2017). A validated liquid chromatography–tandem mass spectrometric (LC-MS/MS) method for the estimation of fulvestrant in human plasma. Orient. J. Chem..

[22] Jolibois J., Schmitt A., Royer B. (2019). A simple and fast LC-MS/MS method for the routine measurement of cabozantinib, olaparib, palbociclib, pazopanib, sorafenib, sunitinib and its main active metabolite in human plasma. J. Chromatogr. B Anal. Technol. Biomed. Life Sci..

[23] Beer B., Schubert B., Oberguggenberger A., Meraner V., Hubalek M., Oberacher H. (2010). Development and validation of a liquid chromatography-tandem mass spectrometry method for the simultaneous quantification of tamoxifen, anastrozole, and letrozole in human plasma and its application to a clinical study. Anal. Bioanal. Chem..

[24] Nalanda R.B., Srinivasa Rao A., Gowri Sankar D. (2018). Determination of palbociclib in human plasma using high performance liquid chromatography—Ultraviolet detection. Int. J. Pharm. Sci. Res..

[25] Leenhardt F., Gracia M., Perrin C., Muracciole-Bich C., Marion B., Roques C., Alexandre M., Firmin N., Pouderoux S., Mbatchi L. (2020). Liquid chromatography–tandem mass spectrometric assay for the quantification of CDK4/6 inhibitors in human plasma in a clinical context of drug-drug interaction. J. Pharm. Biomed. Anal..

[26] Daina A., Michielin O., Zoete V. (2017). SwissADME: A free web tool to evaluate pharmacokinetics, drug-likeness and medicinal chemistry friendliness of small molecules. Sci. Rep..

[27] ChemAxon Chemicalize. www.chemicalize.com.

[28] Phenomenex Strata X-C SPE Products. https://www.phenomenex.com/products/strata-x-solid-phase-extraction-products/strata-x-c.

[29] Phenomenex SPE Method Development Tool. https://www.phenomenex.com/Tools/SPEMethodDevelopment.

[30] Mutavdžić Pavlović D., Babić S., Dolar D., Ašperger D., Košutić K., Horvat A.J.M., Kaštelan-Macan M. (2010). Development and optimization of the SPE procedure for determination of pharmaceuticals in water samples by HPLC-diode array detection. J. Sep. Sci..

[31] Samant T.S., Dhuria S., Lu Y., Laisney M., Yang S., Grandeury A., Mueller-Zsigmondy M., Umehara K., Huth F., Miller M. (2018). Ribociclib Bioavailability Is Not Affected by Gastric pH Changes or Food Intake: In Silico and Clinical Evaluations. Clin. Pharmacol. Ther..

[32] Fujiwara Y., Tamura K., Kondo S., Tanabe Y., Iwasa S., Shimomura A., Kitano S., Ogasawara K., Turner P.K., Mori J. (2016). Phase 1 study of abemaciclib, an inhibitor of CDK 4 and 6, as a single agent for Japanese patients with advanced cancer. Cancer Chemother. Pharmacol..

[33] Tamura K., Mukai H., Naito Y., Yonemori K., Kodaira M., Tanabe Y., Yamamoto N., Osera S., Sasaki M., Mori Y. (2016). Phase I study of palbociclib, a cyclin-dependent kinase 4/6 inhibitor, in Japanese patients. Cancer Sci..

[34] Dowsett M., Cuzick J., Howell A., Jackson I. (2001). Pharmacokinetics of anastrozole and tamoxifen alone, and in combination, during adjuvant endocrine therapy for early breast cancer in postmenopausal women: A sub-protocol of the “Arimidex^TM^ and tamoxifen alone or in combination” (ATAC) trial. Br. J. Cancer.

[35] Desta Z., Kreutz Y., Nguyen A., Li L., Skaar T., Kamdem L., Henry N., Hayes D., Storniolo A., Stearns V. (2011). Plasma Letrozole Concentrations in Postmenopausal Women with Breast Cancer Are Associated with CYP2A6 Genetic Variants, Body Mass Index, and Age. Clin. Pharmacol. Ther..

[36] European Medicines Agency (EMA) EPAR Product Information: Faslodex. https://www.ema.europa.eu/en/documents/product-information/faslodex-epar-product-information_en.pdf.

