# Supplementary material for: Optimisation of Solid-Phase Extraction and LC-MS/MS Analysis of Six Breast Cancer Drugs in Patient Plasma Samples
Source: Pharmaceuticals (Basel). 2023 Oct 12;16(10):1445. doi: 10.3390/ph16101445 (PMC10610126; doi:10.3390/ph16101445)
Supplement: Supplementary file 1 [file pharmaceuticals-16-01445-s001.zip › pharmaceuticals-2644921-supplementary.pdf]

Supplementary materials

Section 1. Chromatograms of blank samples prepared using different SPE sorbents

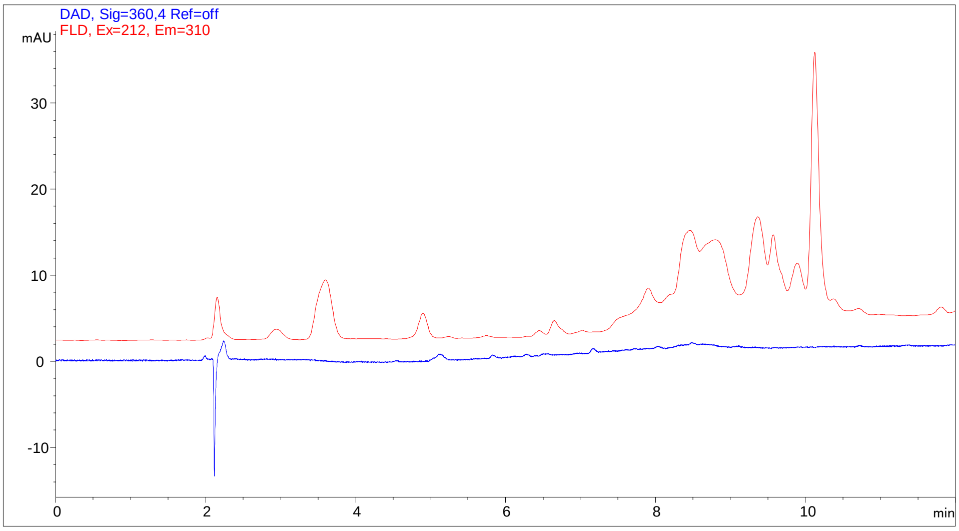

Figure S1. Chromatogram of a blank plasma sample prepared using the Sep-Pak Vac C18 sorbent (200 mg/3 mL) eluted with HCOOH in MeOH. Detection wavelengths: DAD 360 nm (blue), FLD Ex 212, Em 310 nm (red).

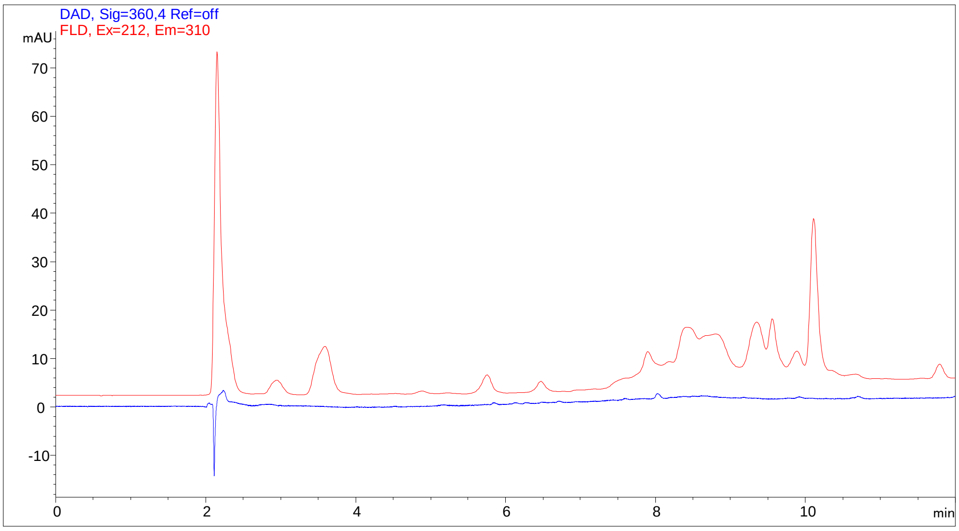

Figure S2. Chromatogram of a blank plasma sample prepared using the Sep-Pak Vac C8 sorbent (200 mg/3 mL) eluted with MeOH. Detection wavelengths: DAD 360 nm (blue), FLD Ex 212, Em 310 nm (red).

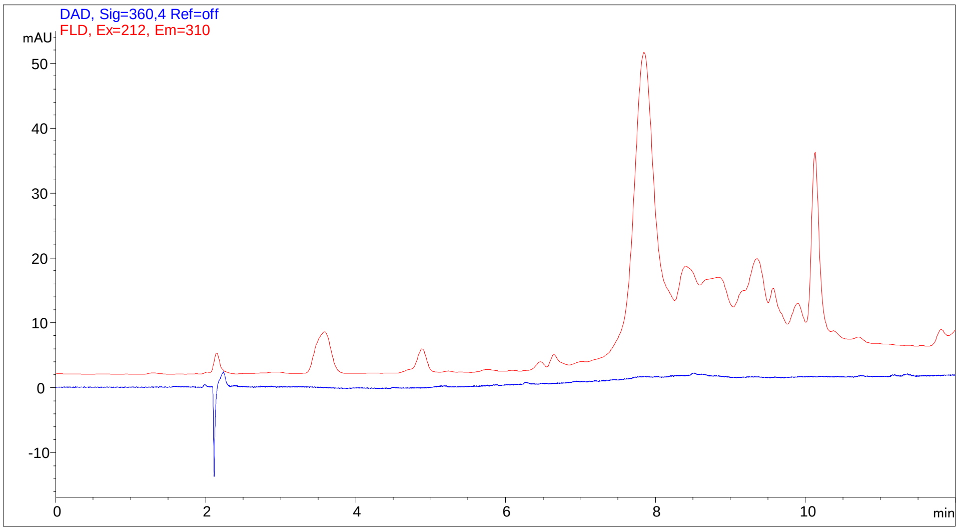

Figure S3. Chromatogram of a blank plasma sample prepared using the Oasis HLB sorbent (60 mg/3 mL) eluted with HCOOH in MeOH. Detection wavelengths: DAD 360 nm (blue), FLD Ex 212, Em 310 nm (red).

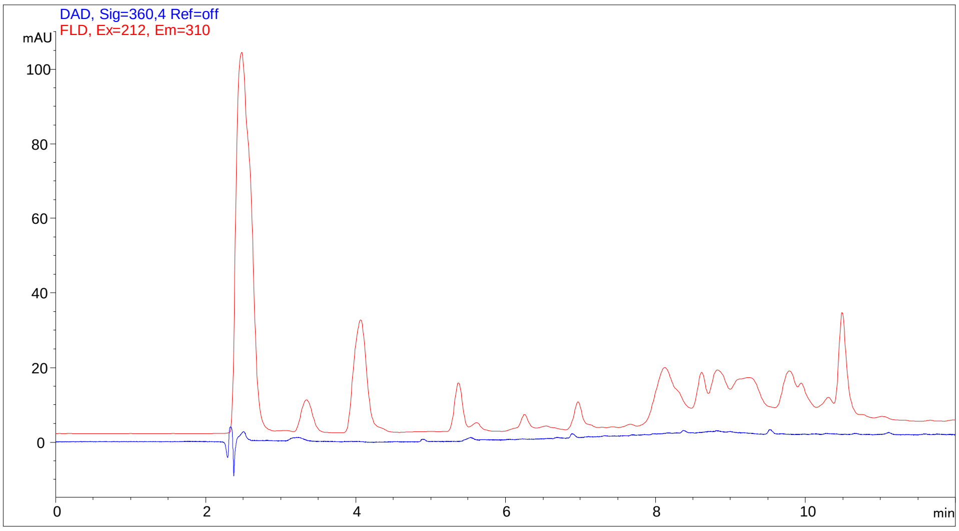

Figure S4. Chromatogram of a blank plasma sample prepared using the Oasis MCX sorbent (30 mg/1 mL) eluted with 5% NH_4OH in MeOH. Detection wavelengths: DAD 360 nm (blue), FLD Ex 212, Em 310 nm (red).

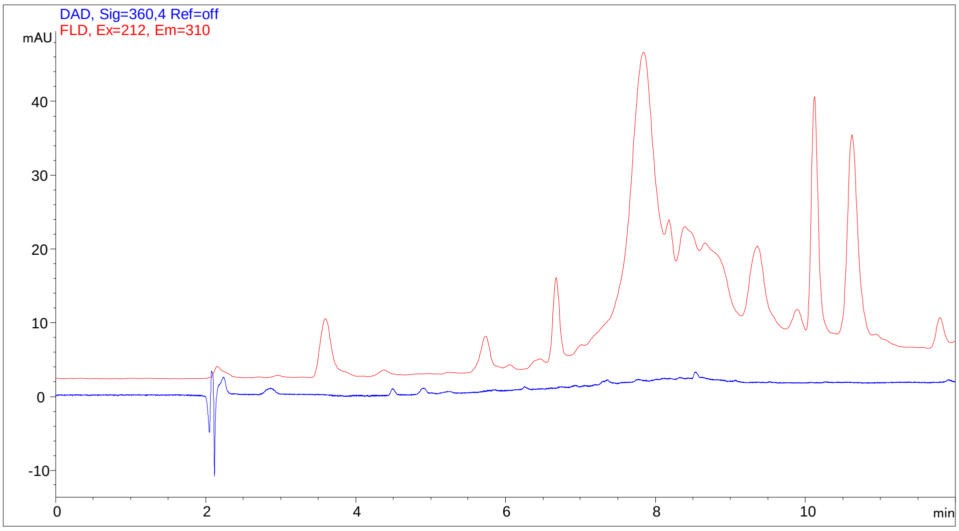

Figure S5. Chromatogram of a blank plasma sample prepared using the Oasis WCX sorbent (60 mg/3 mL) eluted with MeOH. Detection wavelengths: DAD 360 nm (blue), FLD Ex 212, Em 310 nm (red).

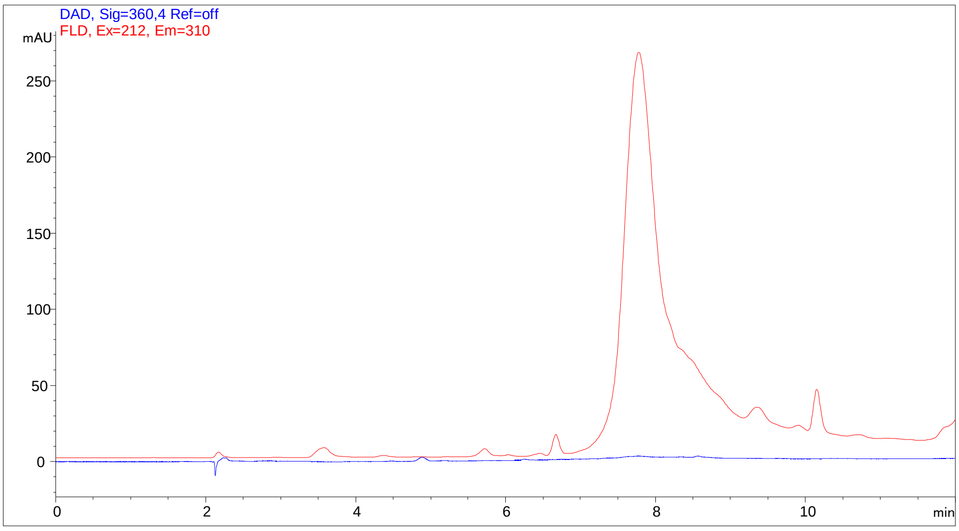

Figure S6. Chromatogram of a blank plasma sample prepared using the Oasis WCX sorbent (60 mg/3 mL) eluted with HCOOH in MeOH. Detection wavelengths: DAD 360 nm (blue), FLD Ex 212, Em 310 nm (red).

Section 2. Method validation

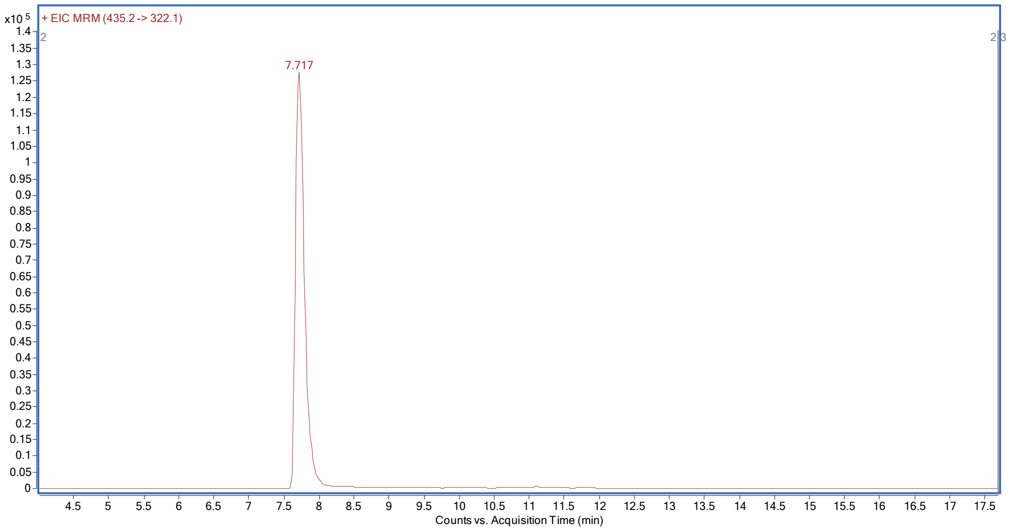

(a)

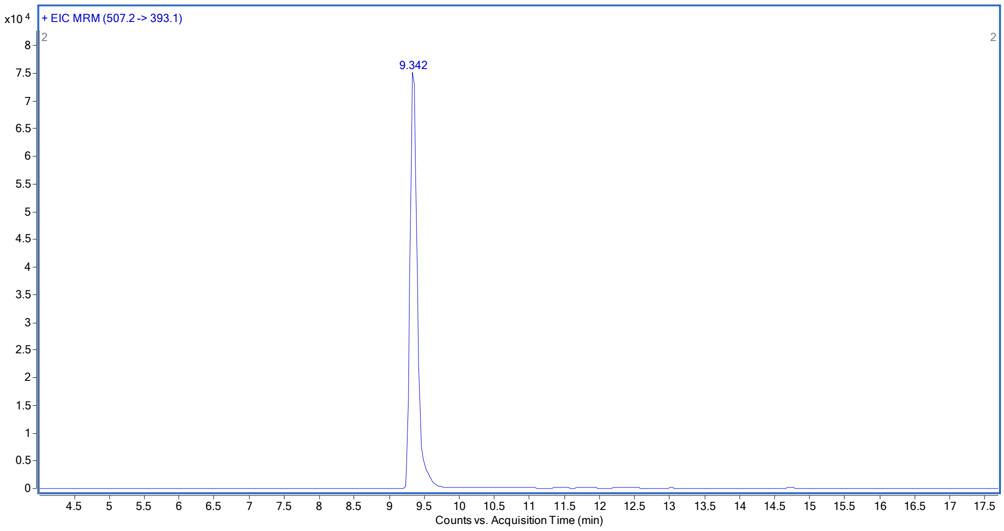

(b)

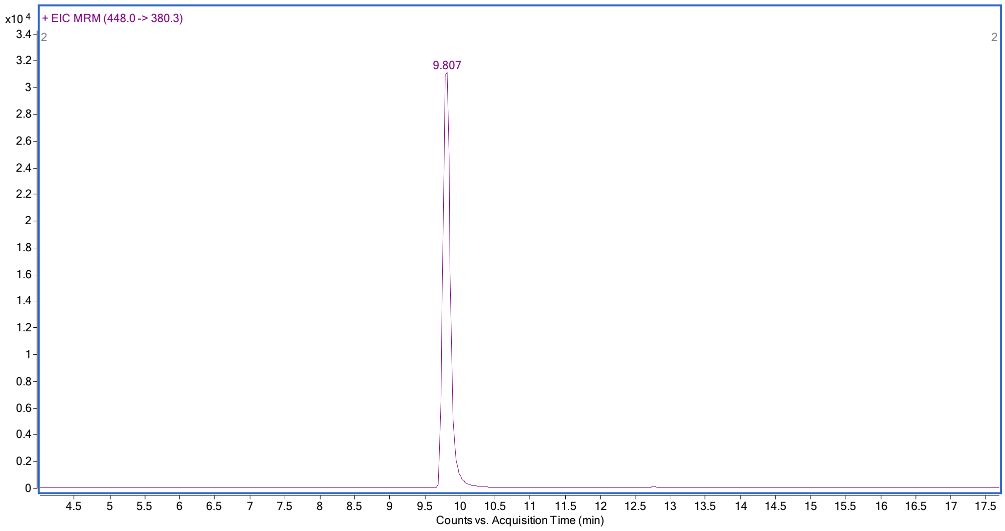

(c)

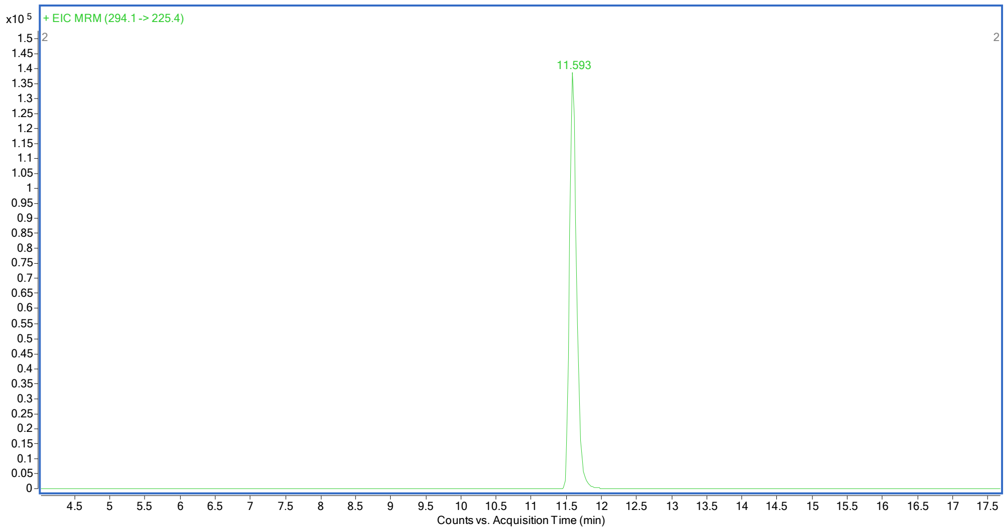

(d)

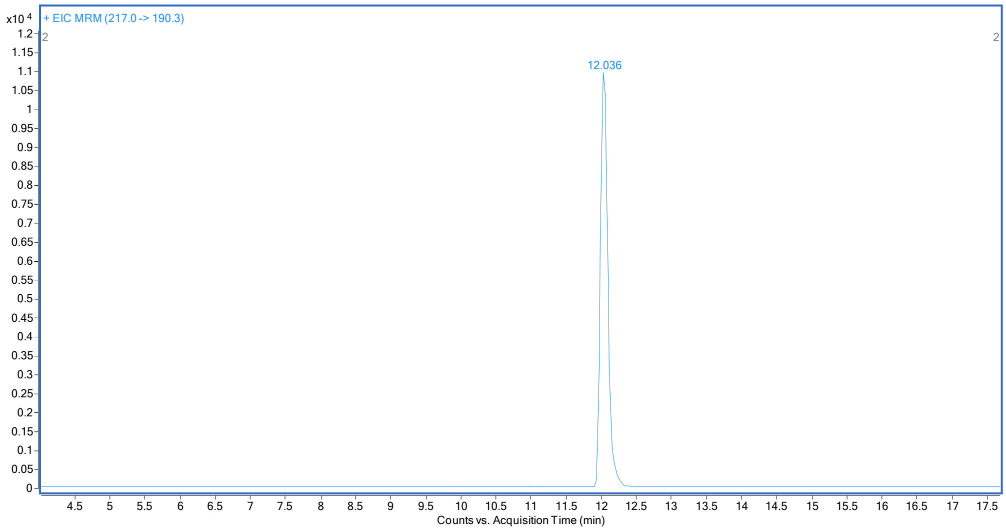

(e)

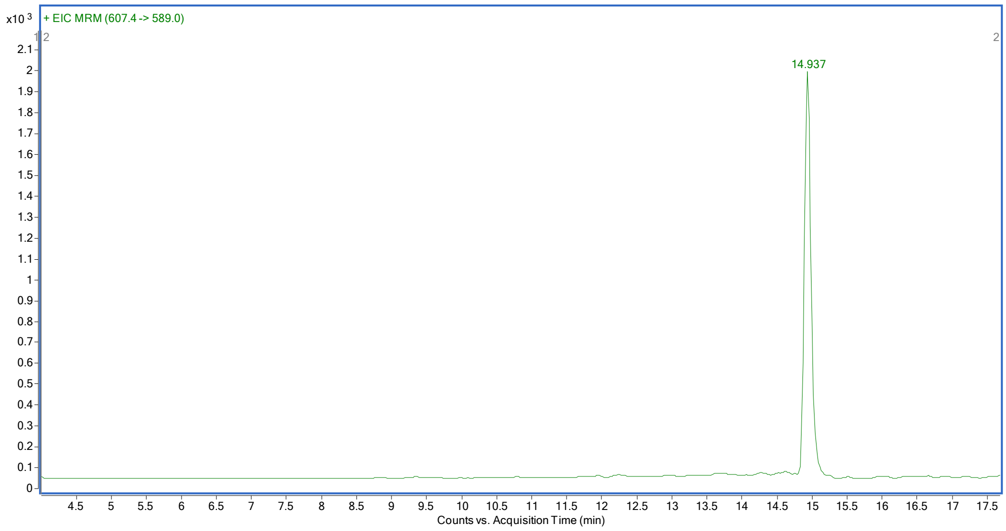

(f)

Figure S7. Extracted ion chromatograms of the quantifier ion transition for the analytes at the LLOQ concentration level: (a) RIB, (b) ABE, (c) PAL, (d) ANA, (e) LET, (f) FUL.

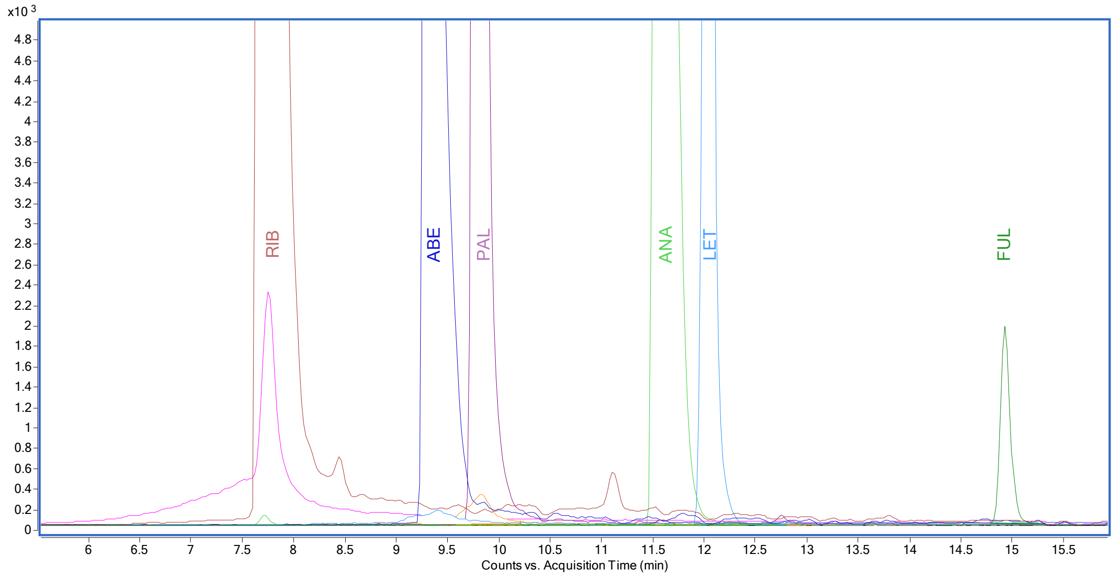

Figure S8. Overlaid extracted ion chromatograms of the quantifying ion transitions for all analytes at the LLOQ concentration levels and in a blank sample injected after the ULOQ concentration level. Brown – RIB, dark blue – ABE, dark red – PAL, light green – ANA, light blue – LET, dark green – FUL.

Table S1. Overall matrix effects results – average value of matrix effects and peak area RSD between all tested samples for each analyte.

Analyte	Concentration (ng/mL)	Matrix effect (%)	Peak area RSD (%)
RIB	1120	14.8	8.6
	2800	8.9	5.2
ABE	128	8.0	11.4
	320	4.0	12.0
PAL	64	−9.0	18.6
	160	−2.3	14.6
ANA	32	−18.7	6.4
	80	−19.5	8.3
LET	64	−56.2	22.2
	160	−57.9	25.2
FUL	16	−29.1	23.4
	40	-33.3	20.7

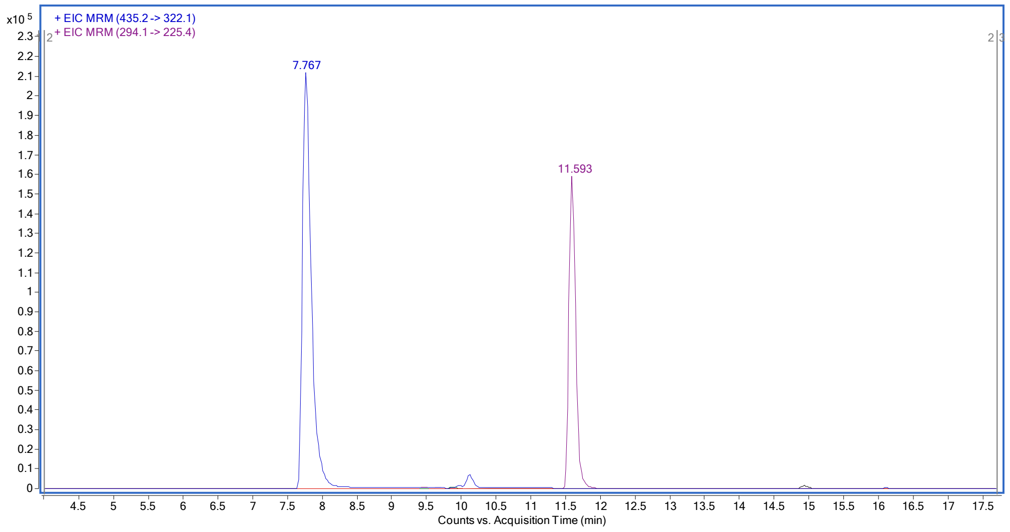
x10 5
+ EIC MRM (435.2 -> 322.1)
+ EIC MRM (294.1 -> 225.4)
7.767
11.593
Counts vs. Acquisition Time (min)

(a)

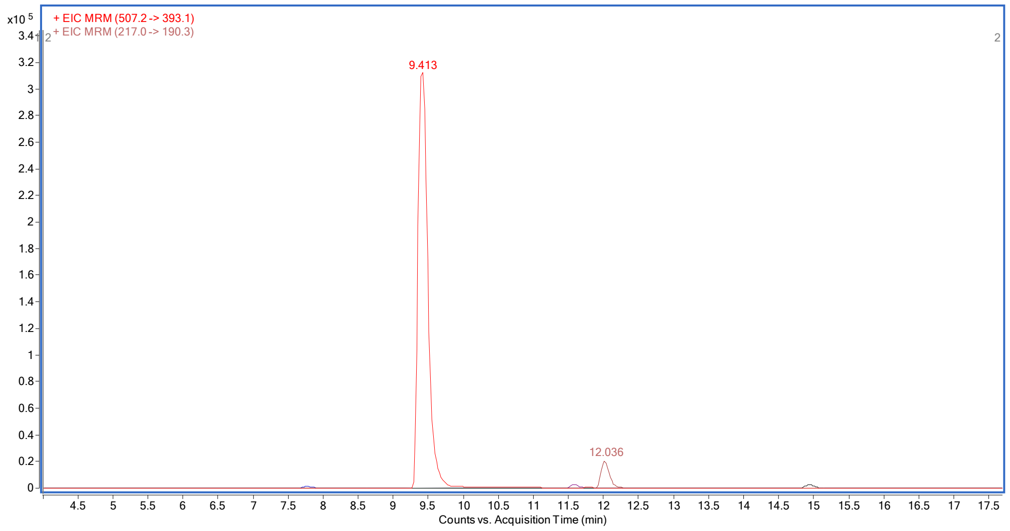
x10 5
+ EIC MRM (507.2 -> 393.1)
+ EIC MRM (217.0 -> 190.3)
9.413
12.036
Counts vs. Acquisition Time (min)

(b)

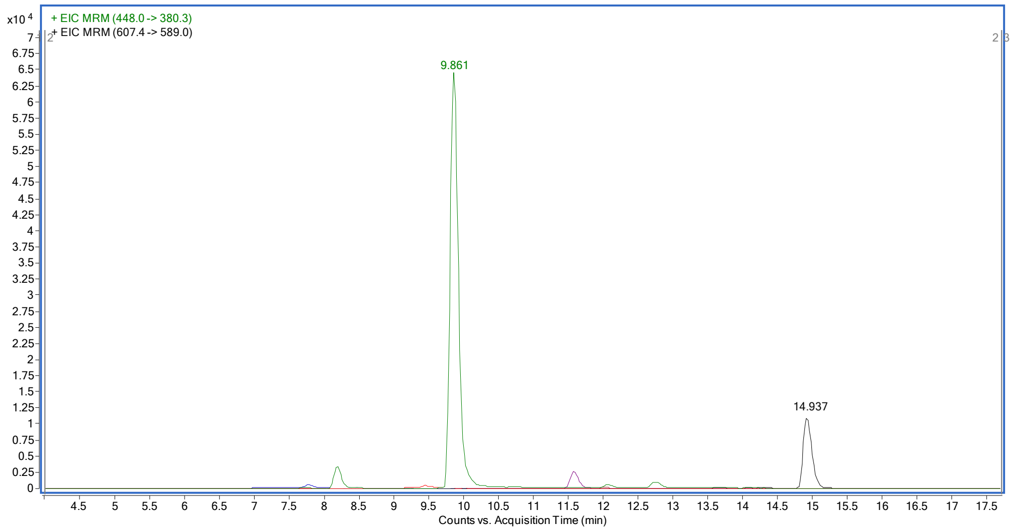
x10 4
+ EIC MRM (448.0 -> 380.3)
+ EIC MRM (607.4 -> 589.0)
9.861
14.937
Counts vs. Acquisition Time (min)

(c)

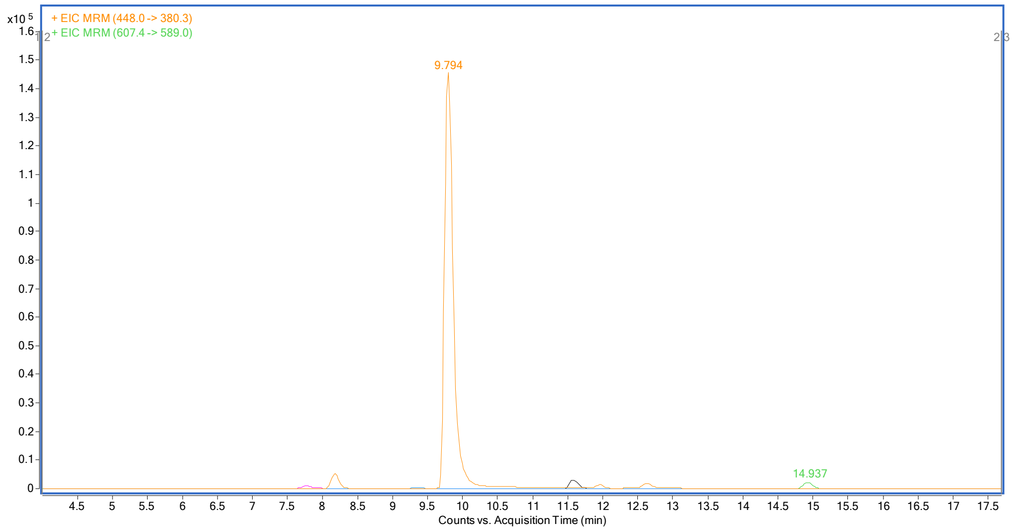

(d)

Figure S9. MS chromatograms of the samples from: (a) patient 1 (RIB, ANA); (b) patient 2 (ABE, LET); (c) patient 3 (PAL, FUL); (d) patient 4 (PAL, FUL).

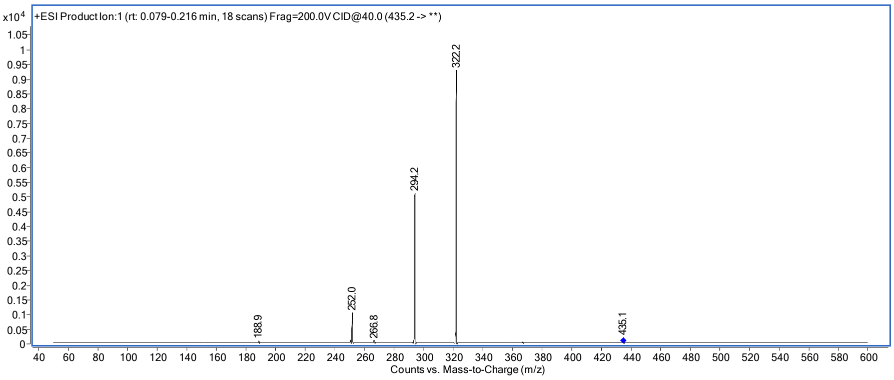

(a)

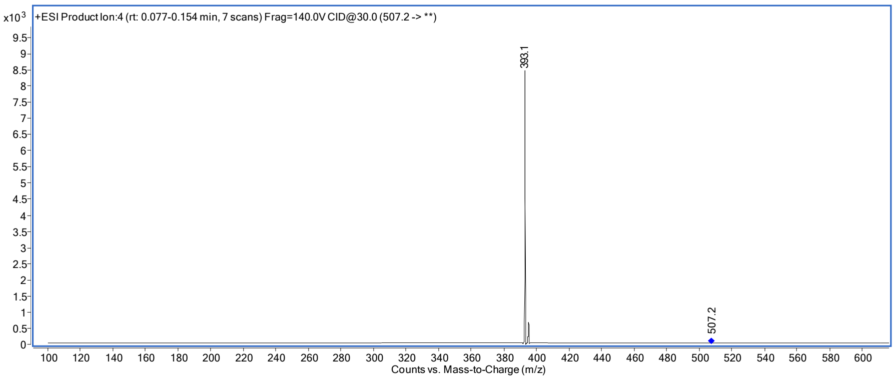

(b)

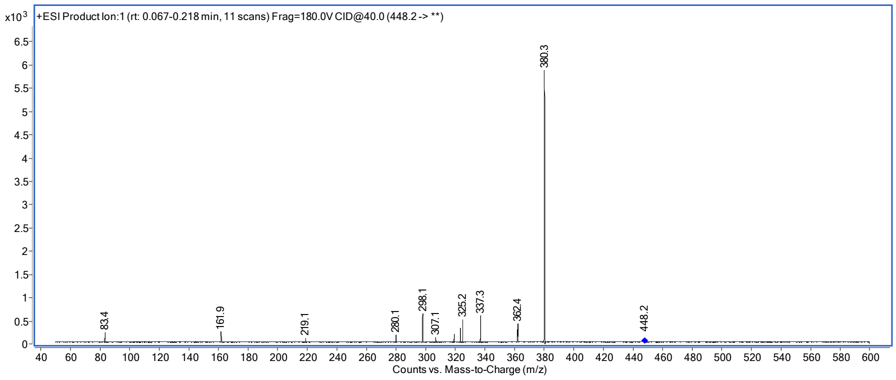

(c)

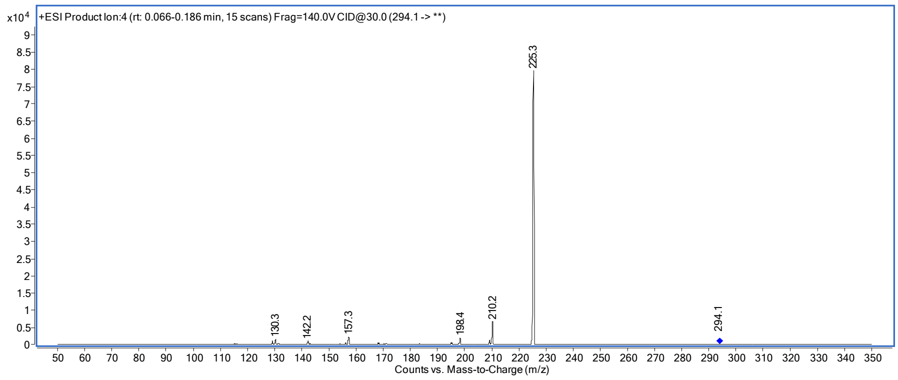

(d)

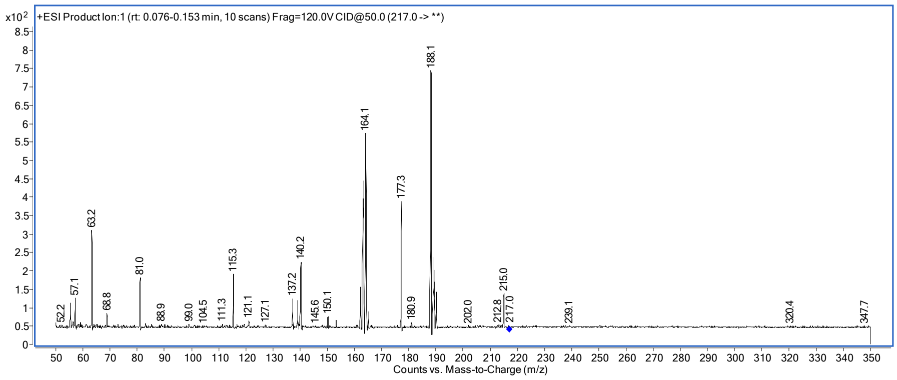

(e)

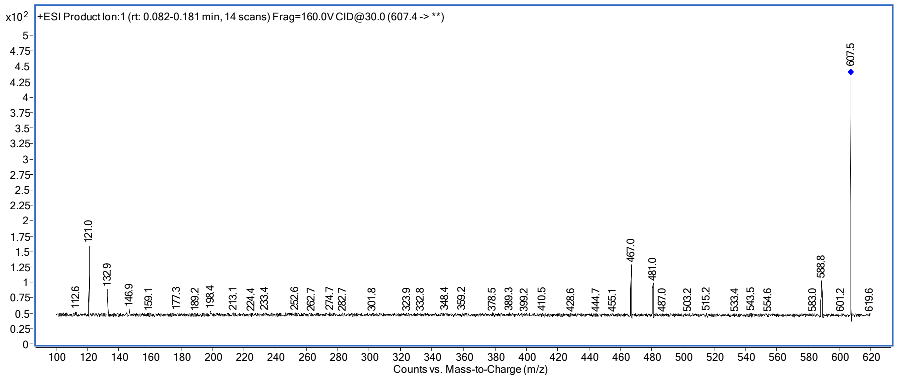

(f)

Figure S10. Exemplary MS spectra of the analytes: (a) RIB (precursor ion *m*/*z* 435.2; CE 40 V, fragmentor 200 V), (b) ABE (precursor ion *m*/*z* 507.2; CE 30 V, fragmentor 140 V), (c) PAL (precursor ion *m*/*z* 448.0; CE 40 V, fragmentor 180 V), (d) ANA (precursor ion *m*/*z* 294.1; CE 30 V, fragmentor 140 V), (e) LET (precursor ion *m*/*z* 217.0; CE 50 V, fragmentor 120 V), (f) FUL (precursor ion *m*/*z* 607.4; CE 30 V, fragmentor 160 V).
